# Supplementary material for: A wind environment and Lorentz factors of tens explain gamma-ray bursts X-ray plateau
Source: Nat Commun. 2022 Sep 24;13:5611. doi: 10.1038/s41467-022-32881-1 (PMC9509382; doi:10.1038/s41467-022-32881-1)
Supplement: Supplementary file 1 — Supplementary Information [file 41467_2022_32881_MOESM1_ESM.pdf]

# A wind environment and Lorentz factors of tens explain gamma-ray bursts X-ray plateau

**Hüsne Dereli-Bégué[1,*], Asaf Pe'er[1], Felix Ryde[2], Samantha R. Oates[3], Bing Zhang[4,5], and Maria G. Dainotti[6]**

[1]Department of Physics, Bar-Ilan University, Ramat-Gan 52900, Israel
[2]Department of Physics, KTH Royal Institute of Technology and The Oskar Klein Centre, SE-106 91 Stockholm, Sweden
[3]School of Physics and Astronomy & Institute for Gravitational Wave Astronomy, University of Birmingham, Birmingham B15 2TT, UK
[4]Nevada Center for Astrophysics, University of Nevada, Las Vegas, Nevada, NV 89154
[5]Department of Physics and Astronomy, University of Nevada, Las Vegas, Nevada, NV 89154
[6]National Astronomical Observatory of Japan, 2-21-1 Osawa, Mitaka, Tokyo 181-8588
[*]husnedereli@gmail.com

## ABSTRACT

This file contains the supplementary information for the paper : A wind environment and Lorentz factors of tens explain gamma-ray bursts X-ray plateau.

## Supplementary Information

### Supplementary Method 1. Sample and data analysis

We use the sample of 222 GRBs with plateau phase and with known redshifts[1] defined in Ref.[1] (see also Refs.[2–5]). These GRBs were detected by the Neil Gehrels *Swift* Observatory[6] from January 2005 until August 2019. They represent the 56% of the GRBs (394) with known redshifts observed by the *Swift* satellite in this period. To define this sample, two criteria are used by Ref.[1], (1) the X-ray light curve of GRBs must have at least one data point in the beginning of the plateau phase and (2) possess a plateau angle smaller than 41°. The choice of the 41° comes from the analysis performed in Refs.[7,8] to allow a more reliable identification of the plateau itself. The plateau phase is identified by fitting the *Swift*-BAT and XRT data together with the phenomenological Willingale model[9].

In order to make our analysis as reliable as possible, we further limit the sample to burst having the best quality observations. Therefore, we added three further criteria to the ones used by Ref.[1]. We require (i) a long lasting plateau phase spanning several thousands of seconds with a temporal X-ray slope larger than $-0.7$, followed by a power-law decay phase at later times (the self-similar phase). (ii) A sufficiently large number of data points ($\gtrsim 5$ which corresponds to the number of parameters used to fit the data with one break) during the plateau and self-similar phases to enable the fits to give well constrained parameter values. For this to be valid, we excluded all X-ray flares (see figure 1 of Ref.[10])[2] defined in the online *Swift* repository[3] from the analyzed data. When, after removing the flares we end up having too few data points either during the plateau or the self-similar phase, we excluded the GRB from the analysis.

[1]https://www.mpe.mpg.de/~jcg/grbgen.html

[2]Flares share many properties with prompt emission pulses. They are most likely linked to the late central engine activities[10,11]. They produce steeper slope during both the plateau and the self-similar phase[12–16]. Moreover, Ref.[17] shows that flares cannot be produced by the forward shock.

[3]https://www.swift.ac.uk/xrt_live_cat/

After applying those criteria, we ended up with 130 GRBs having a well-defined X-ray plateau emission that is followed by a power-law decay phase (which can be interpreted as the late afterglow, or the self-similar phase). Our last criteria is to require an optical counterpart at around the same time as X-ray data. We find 24 GRBs in Ref.[2] consistent with all these criteria. However, we only have full access to the published optical data. Therefore, we perform the analysis and confront our model only to a representative sample made of 13 GRBs listed in Supplementary Tables 1, 2, 3.

### *Supplementary Method 1a. X-ray data and fitting process*

The X-ray count rate light curves (hereinafter LCs)[18] of each 13 GRBs have been downloaded from the online *Swift* repository[4] , for the full *Swift*-XRT bandpass ($E_{\rm min}, E_{\rm max}$) = (0.3,10) keV. We then fit each light-curve with a two or three-segments broken power-law (BPL) model (i.e. with one or two break times) depending on the required break during the late afterglow phase (self-similar phase). The latter break generally coincides with a jet break (see the canonical X-ray afterglow light curve[5] in Ref.[10], figure 1 therein). The X-ray light-curve of 13 GRBs together with our fit results are presented in Supplementary Figs. 1a, 2a, 3a, 4a, 5a, 6a, 7a, 8a, 9a, 10a, 11a, 12a, 13a.

The two-segments BPL model is characterized by the following simple function

$$f(t) = A \times \begin{cases} (t/t_{\rm b})^{-\alpha_1} & \text{for } t < t_{\rm b}, \\ (t/t_{\rm b})^{-\alpha_2} & \text{for } t > t_{\rm b} \end{cases} . \tag{1}$$

In this expression t is the time, $t_{\rm b}$ is the break time, A is the amplitude at the break time, $\alpha_1$ and $\alpha_2$ are the temporal slopes before and after the break time respectively. The three-segments BPL model consider of an additional break and segment.

During the fit, we excluded the early steep decay segment and highly significant emission bumps following the GRB prompt tails (see Ref.[10] figure 1 therein) defined in the online *Swift* repository. In addition, we removed all flares. Some faint flares are not defined in the online *Swift* repository, however, they affect the definition of the break time at the end of the plateau phase ($T_{\rm a}$) in individual GRB light curves. Comparing the optical and the X-ray break times we found that the X-ray break time ($2.8 \pm 0.6 \times 10^3$ s) in the LC of GRB 091029 is earlier than the optical one ($1.54 \pm 0.11 \times 10^4$ s). This is in contrast with the theoretical expectation that, due to the rise in the cooling frequency, the transition time is expected to be earlier in the optical band than in the X-ray band. Therefore, by checking the BAT and XRT unabsorbed flux density light curves at 10 keV retrieved from the online *Swift* burst analyser repository, we identified a flare between $1379 - 2421$s in the X-ray LC of GRB 091029. Then we objectively removed this flare in the X-ray LC of this burst. It is also important to note that the flares in the online *Swift* repository [6] using the phenomenological Willingale model[9] are indicative and not precise.

It is also known that the data obtained by window timing (WT) mode can be effected more from the high latitude emission than the data obtained by photon counting (PC) mode because the WT mode is usually taken at early times as it measures the flux better when the source is bright (see Ref.[19] for more explanation). Light-curves containing both WT and PC data require special attention to analyse the effect of different data type on the slopes. To avoid this issue, we only used data acquired in PC mode even if WT mode data exist (namely for GRB 061121 and GRB 060605). Once the data were retrieved and reduced, we perform a fit to determine the X-ray temporal slopes ($\alpha_{\rm p,X}$, $\alpha_{\rm A1,X}$, $\alpha_{\rm A2,X}$) and the time of the transition between the two components, $T_{\rm a,X}$ as well as the time of the jet break $T_{\rm b,X}$.

[4] https://www.swift.ac.uk/xrt_curves/

[5] The canonical X-ray afterglow light curve shows five distinct components: the steep decay phase, which is the tail of prompt emission; the shallow decay phase (or plateau); the normal decay phase; the late steepening phase; X-ray flares.

[6] https://www.swift.ac.uk/xrt_live_cat/docs.php

**An example of the fitting process:** To visualise the fitting process, we illustrate in Supplementary Fig. 3a the X-ray count light-curve obtained from GRB 060614 by the *Swift*-XRT instrument (over the period of PC mode data acquisition). The data are superposed with the fits results obtained for a three-segments BPL model, i.e. with two break times. To perform the fit, we used the Bayesian analysis tool *emcee*[7]. We set up the computation with 64 MCMC walkers, a 100 burn-in period and the 5000 MCMC steps. We chose these steps taking into account the auto-correlation time. The value of the MCMC steps can slightly change for each GRB depending on the auto-correlation time but the choice of 5000 steps is the one that guarantees that the auto-correlation time is always taken into account. We employ the following uninformative prior distributions for the fit parameters

$$\begin{cases} Pr(N) = \mathscr{U}(-5,5), \\ Pr(\alpha_{\rm p}) = \mathscr{U}(-5,5), \\ Pr(\alpha_{\rm A1}) = \mathscr{U}(-5,5), \\ Pr(\alpha_{\rm A2}) = \mathscr{U}(-5,5), \\ Pr(\log(T_{\rm a})) = \mathscr{U}(2,4.5), \\ Pr(\log(T_{\rm b})) = \mathscr{U}(4.5,6), \\ Pr(\log(V)) = \mathscr{U}(-15,12), \end{cases} \tag{2}$$

where $N$ is the normalisation, $\alpha_{\rm p}$, $\alpha_{\rm A1}$ and $\alpha_{\rm A2}$ are the slopes during the plateau, in the self-similar phase and following the jet break respectively. Here, $\mathscr{U}$ notes the uniform prior. The characteristics times $T_{\rm a}$ and $T_{\rm b}$ mark the end of the plateau and the jet break respectively. Finally $V$ is a nuisance parameter measuring the spread of the data. The parameter range for the normalisation can change depending on the band (X-ray or optical) in which the fit is performed.

As a Specific example, we show the fit parameters of GRB 060614 in Supplementary Table 4. The corner plot of the posterior probability distributions of the fit parameters and the covariances between the fit parameters is displayed in Supplementary Fig. 14.

The temporal fit parameters in the X-ray band obtained for all 13 GRBs in our sample are presented in Supplementary Tables 1, 2, 3, for different classes I, II, and III respectively. The definitions of these classes are explained in Results subsection Sample classification in the main manuscript.

The X-ray spectral slopes ($\beta_{\rm p,X}$, $\beta_{\rm A1,X}$, $\beta_{\rm A2,X}$) of twelve GRBs out of thirteen (all GRBs but GRB 060729) for the time intervals defined by the characteristic times of the X-ray light-curve are also obtained from the online *Swift* repository and presented in Supplementary Tables 1, 2, 3. It is important to note that the photon index (which is equal to $\beta+1$ where $\beta$ is the spectral index) is presented in the online *Swift* repository. For GRB 060729, we used the result from the spectral analysis of Ref.[21] since the value of the spectral index is not given by the online *Swift* repository. These X-ray spectral and temporal parameters together with temporal slopes obtained from the temporal fit are combined into the closure relations ($F_\nu \propto t^{-\alpha}\nu^{-\beta}$) as explained in the Result subsection Closure relations and determination of the electron power-law indices. This relations are presented in Supplementary Figs. 15 and 16.

**Caveats for the X-ray analysis for specific GRBs:**

- GRB 080607. For this burst, the error of the temporal slope during the plateau phase is large. This is due to the lack of the data points at the end of the X-ray plateau phase. The slope after the plateau

[7] *emcee* is an MIT licensed pure-Python implementation of Goodman & Weare's Affine Invariant Markov chain Monte Carlo (MCMC) Ensemble sampler[20]

phase is rather steep. However, the later (last) slope is more compatible with the expectations from the self-similar phase, see, Supplementary Fig. 8a. Therefore, we used later slope presented in Supplementary Table 1. The light-curve has two flares before the onset of the plateau phase. The transition between the peak-flux of the second flare and the plateau phase is well represented by a BPL, with steep slopes $-2.27^{+0.07}_{-0.07}$ and $-1.42^{+0.25}_{-0.30}$, separated at a break time of $341^{+31}_{-28}$s. The post-flare slopes are similar to the slopes found after the plateau phase. Therefore, either another flare might exist during the plateau or the plateau slope might be steeper with a break later than the $T_{\rm a,X}$ obtained here.

- GRB 130831A: The lack of data in the X-ray LC of this GRB between $10^3$ and $10^4$s, typical for the duration of the plateau, affects the measurement of $T_{\rm a,X}$, see Supplementary Fig. 12a. We tried to fit the light curve with a model composed of three power-law and two breaks. For this model, we found that $\log(T_{\rm a,X}/{\rm s}) = 3.08^{+0.28}_{-0.18}$. This break is consistent with being achromatic in both X-ray and optical bands (see, Supplementary Method 1b, Optical data and fitting process). However, due to the large uncertainty on $T_{\rm a,X}$ we found that fitting the light curve with a single break provides a better fit. Therefore, in our analysis we used the parameters obtained for the model with one temporal break. These parameters are given in Supplementary Table 1. In addition, before the lack of data, we also define a faint flare between $595-811$ s in the X-ray LC of this burst. This flare coincides with the beginning of the optical flare, see Supplementary Fig. 12b. Therefore, we also performed a fit without this flare. We found that the both slopes ($-0.80^{+0.03}_{-0.03}$ and $-0.98^{+0.06}_{-0.06}$ respectively with a break at $1.0^{+0.25}_{-0.16}\times 10^4$ s) are consistent with the expected slope ($\sim -1.2$) in a self-similar phase. This result is also consistent with a previous study in Ref.[22]. We then conclude that either this GRB is not the best representation of the GRBs that show plateau phase or the lack of the data is misleading the results.

### *Supplementary Method 1b. Optical data and fitting process*

Below, we describe the details of the fitting procedure in the optical band for each GRB in our sample. Before making the fits and if needed, the data were first transformed to the AB magnitude system. The results of the fits (in the temporal regions of interest) are presented in Supplementary Tables 1, 2 and 3. The following detailed explanations for each GRB independently are split depending on the origin of the data.

For GRB 091029, GRB 110213A, GRB 130831A and GRB 171205A, the optical data were retrieved from all possible published sources listed in Supplementary Table 1. For each of these bursts, we compiled a large set of data obtained in different bands from different instruments. The temporal fits are performed simultaneously in each band, which means that we consider all the bands at the same time, only changing the normalisation between the observation bands. Our aim here is to reduce the dependence on a single instrument and a single band in the determination of the temporal slopes and the break times. This method provides tight estimations of the fit parameters.

- GRB 091029: The photometric data set was retrieved from tables 5, 6, 7, 8, 9 of Ref.[23]. The data are displayed alongside our fit results in Supplementary Fig. 9b. We fit the data between 427.7s and $2\times 10^5$s with a three-segment broken power law model (i.e. with two breaks, hereinafter two-break model). Although later data exist, the flattening in the U-band likely indicates an external source of radiation, such as the contribution from the host galaxy. Therefore, data after $2\times 10^5$s are excluded

from the fit. The first temporal break at $3.7^{+0.3}_{-0.2} \times 10^3$s is interpreted as the beginning of the plateau, although the earlier (i.e. before the plateau) slope is $-0.60^{+0.02}_{-0.01}$.

- GRB 110213A: The photometric observations are obtained from table 4 of Ref.[24]. They are displayed alongside our fit results in Supplementary Fig. 11b. The optical light-curve of this GRB has two peaks. The brightest peak is at 263s and the second peak is at 4827s. This flare can be clearly seen in the unabsorbed flux density light curve (see *Swift*-BAT-XRT at 10 keV in the online *Swift* burst analyser repository). This flare produces a rising slope during the X-ray plateau phase, however, it has a negligible effect on the value of break time at the end of the plateau phase $T_{\rm a,X}$.

- GRB 130831A: The photometric data of this burst is obtained from the VizieR Online Data Catalog[25]. The data are displayed alongside our fit results in Supplementary Fig. 12b. A flare can clearly be identified between 425s and 3400s, therefore, this time interval is not considered in our fit. The flare interpretation of this re-brightening is further supported by the fact that we found the temporal slopes before 425s and after 3400s to be the same. The light curves in the Rc and Ic bands flatten after $10^5$s, most likely due to an external source. Shortly later, this flattening also appears in all other bands. Therefore, the data after $10^5$s are discarded from our analysis.

- GRB 171205A: The photometric data set is obtained from Ref.[26]. The data set is displayed alongside our fit results in Supplementary Fig. 13b. Since this GRB is associated to the supernovae SN 2017iuk, the optical data start to rise at $T_{\rm b,O} = 1.73 \times 10^5$ s. We fit all the data before the peak of the supernovae (SN) at $9.51 \times 10^5$ s with a two break model. The first break ($T_{\rm a,O}$) represents the transition from the plateau to the self-similar phase, while the second break, at $T_{\rm b,O}$ marks the rise of the optical light curve due to the supernovae contribution. We find that the temporal slopes are somewhat shallower, and even slightly rising than in other bursts. Moreover, the break time $T_{\rm a,O}$ occurs earlier in the optical band than the X-ray band. We believe that this may be due to the SN bump after $\approx 2$ days[26].

For GRB 050319, GRB 060605, GRB 080607 and GRB 100418A, we used the optical data from table 8 of Ref.[21]. In that paper, all available data obtained from different instruments were collected. The details of the fitting process is given below for each of these GRB.

- GRB 050319: We use a two-break model to fit the optical light-curve until the observer time $t = 5.34 \times 10^5$ s, after which the observations show a steep flux decrease with an exponent steeper than $-1.2$ (which is theoretically expected from the self-similar phase.) The first break is observed at time $371^{+31.6}_{-28.6}$ s, which is too small to be the end of the plateau in the framework of our model. We therefore consider the break at $3.58^{+0.30}_{-0.32} \times 10^4$ s to be the end of the plateau. In any case, the optical slopes of each phase are not very different: the early (before the plateau) slope is $-0.41^{+0.02}_{-0.01}$, the slope of the plateau is $-0.59^{+0.01}_{-0.01}$ and the slope during the self-similar expansion is $-0.64^{+0.02}_{-0.01}$.

- GRB 060605: We fit the data between 185 s and $2 \times 10^4$ s with with a two-break model. After the time $t = 2 \times 10^4$ s, the optical data flattens in all bands, see, Supplementary Fig. 2b. Therefore, this data is not included in the fit.

- GRB 080607: The LC of this has three breaks at around 89 s, at $793^{+77}_{-64}$ s and at $T_{\rm a,O} = 2.55^{+0.26}_{-0.12} \times 10^3$ s, see Supplementary Fig. 8b. Since we are interested in the late time evolution, we fit the data starting at 89 s. The temporal slope before the plateau phase is $-1.22^{+0.01}_{-0.02}$, which is similar to the

slope of the self-similar phase, see Supplementary Table 3. Contrary to the X-ray band (see above, Supplementary Method 1a, X-ray data and fitting process), the plateau phase in the optical band is clearly identified and characterized.

- GRB 100418A: In our analysis of the optical data, we excluded the data only reported in the GCN (Gamma-ray Coordination Network). For this burst specifically, we fit a one-break model to the data.

The optical LC of the rest of the GRBs in our sample (namely GRB 060614, GRB 060714, GRB 060729, GRB 061121, and GRB 080310) are taken from Ref.[27] (see also, Ref.[28] for the details). These data were obtained by the *Swift* Ultraviolet/Optical (UVOT) instrument in bands V, B, U, W1, M2 and W2. In Ref.[27], all the LCs were normalized to the U band, and we use these normalised LCs in our analysis. The details of the fitting process are given below for each GRB.

- GRB 060614: We fit the data between $4.84 \times 10^3$ and $8.39 \times 10^5$ s. Indeed, at early times between 113.16s and 379.87s, the LC has a steep decay which coincides with the steep decay observed in X-ray data, see Supplementary Fig. 3a. This decay is therefore interpreted as the end of the prompt phase. Moreover, no observations were performed between 380s and $4.8 \times 10^3$s. At times larger than $8.39 \times 10^5$s the LC flattens which is interpreted as host galaxy contribution, see Supplementary Fig. 3b.

- GRB 060714: The observations of this burst are scarce with large errors. We excluded the last data point at $1.49 \times 10^7$s from the fit since it is a clear outlier. We also did not included the non-detection limits in our analysis. With 8 data points left, a break can clearly be identified and the parameter of a one-break model be constrained, even though the observations after $10^5$s have large errors, see Supplementary Fig. 4b.

- GRB 060729: We fit the data between 266s and $1.12 \times 10^6$s. At earlier times, the optical flux rises, and at later times it flattens with a break at $\log(t) = 6.16^{+0.02}_{-0.03}$, while the slope after the break is $-0.16^{+0.07}_{-0.07}$, see Supplementary Fig. 5b.

- GRB 061121: We fit the data between $t = 104.97$ and $1.66 \times 10^5$ s. At early time between 69.19s and 89.97s, the LC has an optical flare which coincides with the X-ray flare defined in the online *Swift* repository. At later times, the data flattens. We further exclude the early optical bump seen between 143.45 and 568.48 s to have a better identification of the plateau slope and the break time $T_{a,O}$, see Supplementary Fig. 6b. If our fit would include the decay of this bump (namely, starting at 230 s), its fitted slope and the break time would be $-1.01^{+0.05}_{-0.06}$ and $\log(T/s) = 3.12^{+0.06}_{-0.06}$ respectively. However, we would find that the later slopes ($-0.45^{+0.06}_{-0.05}$, $-0.93^{+0.07}_{-0.08}$ respectively) and the later break time ($\log(T_a/s) = 4.08^{+0.10}_{-0.10}$) would be compatible with the fit parameters presented in Supplementary Table 3. Therefore, the effect of removing the second optical bump from the analysis is negligible.

- GRB 080310: The first data point at 132.39s has a much lower flux than during the plateau. This data point can be part of the rise at early time. Such a rise is clearly seen in some of the optical LC in our sample (e.g., GRB 060605). Therefore, it is discarded from our analysis. Moreover, we also did not include in our analysis the observation with no detection, see, Supplementary Fig. 7b.

The optical spectral slopes ($\beta_{p,O}$, $\beta_{A1,O}$, $\beta_{A2,O}$) of twelve GRBs out of thirteen in our sample (all except GRB 130831A which does not have a published one) are obtained from the literature: data of GRBs 050319, 060605, 060614, 060729, 061121, 080310, 080607 and 100418A are presented in Ref.[21]; GRB060714 in Ref.[29]; GRB091029 in Ref.[23]; GRB110213A in Ref.[24]; and GRB171205A in Ref.[26]. All these slopes are given in Supplementary Tables 1, 2, 3 for the different classes I, II, III respectively. These spectral slopes are also combined into the closure relation ($F_\nu \propto t^{-\alpha}\nu^{-\beta}$) in Supplementary Figs. 15 and 16 together with temporal slopes obtained from the optical temporal fit. This relation is as explained in the Result subsection Closure relations and determination of the electron power-law indices.

### *Supplementary Method 1c. Flux Ratio*

**X-ray energy flux ($\nu F_\nu$):** The unabsorbed flux density ($F_\nu$) light curves of XRT at 10 keV[19] are obtained from the online *Swift* burst analyser repository[8]. These flux density LCs are already corrected for absorption from both the galactic and host galaxy. In our analysis, we convert these light curves from flux density (Jy) to energy flux (erg cm$^{-2}$ s$^{-1}$) at 10 keV ($\nu = 2.42 \times 10^{18}$ Hz). Then, we used these energy flux LCs to compute the $\nu F_\nu$ X-ray flux at the end of the plateau phase at $T_{a,X}$ and at another specific time, typically, 1000s.

**Optical energy flux ($\nu F_\nu$):** For computing the energy flux in the optical band, all the LCs are corrected for both the galactic and the host galaxy extinctions. i) The galactic extinction along the line of sight is obtained from the IRSA webpage[9] for the U band filter. It is computed with the method introduced in Ref.[30]. ii) The extragalactic reddening E(B - V) of all GRBs (except GRBs 060714 and 100418A) are obtained during the plateau phase and the references are listed in Supplementary Table 5. We first used these values to compute the host galaxy extinctions in the V-band (except for 060714 for which we used the value provided in Ref.[31]). The host galaxy extinction $A_{\nu,\mathrm{host}} = R_\nu \times E(B-V)$ is computed by using one of the three visual extinction to reddening ratio, $R_\nu$, of 3.08, 3.16, 2.93 for the Milky Way, Large and Small Magellanic Clouds (MW, LMC and SMC) extinction laws[32] respectively. The type of extinction law is defined in the reference papers and they are also given in Supplementary Table 5. Second, we use these extinctions together with $A_{\mathrm{u,host}} = 1.666 \times A_{\mathrm{v,host}}$ see, details in Ref.[32], to compute the host galaxy extinction in the U band. Both extinctions (galactic and host galaxy) for each GRBs are given in Supplementary Table 5. Note that the flux density of four GRBs (namely GRB 050319, GRB 060605, GRB 080607, GRB 100418A) taken from Ref.[21] are already corrected for both galactic and host extinction.

In details, we proceed as follows:

- For six GRBs, namely GRB 060614, GRB 060714, GRB 060729, GRB 061121, GRB 080310 and GRB 091029, the optical count rate LCs from Ref.[27] are used to compute the U-band energy flux LCs. For the conversion, we used the U-band count rate to flux conversion factor ($1.628 \times 10^{-16}$ erg cm$^{-2}$ Angstrom$^{-1}$ counts$^{-1}$), as provided in the UVOT calibration website[10]. Then, we computed the energy flux ($\nu F_\nu$) LCs, where $\nu$ is taken at the U-band central frequency equal to $8.65 \times 10^{14}$ Hz. These U-band energy flux LCs are used to compute the $\nu F_\nu(\mathrm{U})$ optical flux at a specific time namely $\sim$ 1000s hereinafter dubbed reference time in the U-band $T_{\mathrm{ref.,U}}$ and at the end of the plateau phase at $T_{\mathrm{a,X}}$.

For all other GRBs, we followed a different procedure presented below:

[8] https://www.swift.ac.uk/burst_analyser/
[9] https://irsa.ipac.caltech.edu/applications/DUST/
[10] https://heasarc.gsfc.nasa.gov/docs/heasarc/caldb/swift/docs/uvot/index.html

i) For the GRBs in Ref.[21], namely GRB 050319, GRB 060605, GRB 080707, and GRB 100418), the $\nu F_\nu$ optical flux at the end of the X-ray plateau phase ($T_{\rm a,X}$) are retrieved from the flux density LC in table 7 of the online material in Ref.[21]. In these cases, the frequency $\nu$ is different for each GRBs (V band $\nu = 5.48\times10^{14}$ Hz, CR and RC bands $\nu = 4.55\times10^{14}$ Hz, white band $\nu = 7.79\times10^{14}$ Hz, see Supplementary Table 5). For consistency, whenever possible we used the U-band in calculating the LC. When the U-band data was not available, we used other bands. In such case, we note that, the differences between different optical bands ($\sim 10^{15}$ Hz) are small.

ii) For all other, namely GRB 110213A, GRB 130831A, and GRB 171205A, we followed the same procedure as in appendix A of Ref.[21] to convert the optical data from the AB magnitude system to the optical energy flux. Therefore, the final conversion formula is $f_\nu = 10^{-0.4(m_{AB}+48.585)}$ where $f_\nu$ is the flux density in frequency, $m_{AB}$ is magnitude in AB system.

**Energy flux ratio:** The specific energy fluxes $\nu F_\nu(\mathrm{X})$ at $T_{\rm a,X}$ in the X-ray band and $\nu F_\nu(\mathrm{U})$ at $T_{\rm ref.,U}$ in the optical band are used to compute the energy flux ratio between the X-ray and the optical bands. The ratio is computed by $[1/(\nu F_\nu(\mathrm{U})/\nu \mathrm{F}_\nu(\mathrm{X}))]$ and is given in the last column of Table 3 in the main manuscript.

**Isotropic energy:** The *Swift*-BAT observations are carried in a relatively narrow energy range (15–150 keV) which prevents from measuring the peak energy. To obtain a lower limit on the isotropic energy $E_{\rm iso}$, we followed the same method presented in Ref.[33]. In this method, a simple power-law spectral model is adopted, i.e. $\phi(E)\propto E^{\alpha_\gamma}$, where $\phi(E)$ is the source photon spectrum and $\alpha_\gamma$ is the photon spectral index. The estimate on the isotropic energy in the BAT band $E_{\rm iso}$ is then computed as

$$E_{\rm iso} = \frac{4\pi d_{\rm L}^2 S}{(1+z)^{3-\alpha_\gamma}}. \tag{3}$$

where $S$ is the BAT fluence between 15 and 150 keV. Both $S$ and $\alpha_\gamma$ are taken from the *Swift* GRB table[11] and presented in Supplementary Table 6 together with the burst duration $T_{90}$ which is obtained from the *Swift* GRB table as well. $d_{\rm L}(z)$ is the luminosity distance calculated assuming a flat $\Lambda$CDM cosmological model with cosmological parameters $\Omega_m = 0.286$ and $H_0 = 70$ km s$^{-1}$ Mpc$^{-1}$, and $z$ is the redshift.

The various parameters relevant to our study are listed in Table 2 (in the main manuscript): (i) the GRB name, (ii) its redshift ($z$), (iii) isotropic energy ($E_{\rm iso}$) in the *Swift*-BAT band ($15-150$ keV), (iv) the time at the end of the X-ray plateau phase ($T_{\rm a,X}$), (v) $\nu F_\nu$ X-ray flux and (vi) $\nu F_\nu$ optical (U band) flux at $T_{\rm a,X}$, (vii) a reference time in the optical band called $T_{\rm ref.,U}$ (typically at around 1000 s), (viii) $\nu F_\nu$ X-ray flux and (ix) $\nu F_\nu$ optical (U band) flux at the reference time $T_{\rm ref.,U}$. The isotropic energy ($E_{\rm iso}$) and a time at the end of the X-ray plateau phase ($T_{\rm a,X}$), firstly discovered by Ref.[34–36], are presented in Fig. 1 (in the main manuscript).

## Supplementary Method 2. Theoretical model

The key to understanding the observed signal within the framework of our model is the realization that the end of the plateau corresponds to the transition from a coasting phase to a self-similar (decaying) expansion phase of the expanding plasma. The observed signal originates entirely from ambient electrons collected and heated by the forward shock wave, propagating at relativistic speeds inside a wind (decaying density) ambient medium. During the transition from the coasting to the self-similar phases a reverse shock crosses the expanding plasma. However, the contribution from electrons heated by the reverse shock

[11] https://swift.gsfc.nasa.gov/archive/grb_table/

is suppressed due to (i) the declining ambient density which implies that the ratio of plasma density to ambient density remains constant (in a conical expansion), and (ii) its slower speed, which translates into less energetic electrons that emit at much lower frequencies than forward shock heated electrons, implying that the contribution to the optical and X-ray bands is negligible.

To understand how this transition affects the observed spectra, we first describe the radiative mechanism adopted, which is the classical synchrotron emission from a power-law distribution of electrons, injected into the radiation zone with a power-law index $p$, namely $N_{el}(\gamma)d\gamma \propto \gamma^{-p}$ above a minimum value $\gamma_m$. Both theory[37] and observations[38] suggest a typical value of $p$ such that $2.0 \leq p \lesssim 2.4$. Below the injection Lorentz factor $\gamma_m$, it is safe to assume that the electrons have a Maxwellian (or quasi-Maxwellian) energy distribution[39,40]. This particle distribution leads to a broken power-law spectrum, whose shape, in the relevant observed bands (frequency $\nu$), depends on whether the peak frequency, $\nu_m$ (defined in Supplementary Equation (6)) is above or below the cooling frequency, $\nu_c$ (defined in Supplementary Equation (7)). At low frequencies, one needs to consider the self-absorption process, whose contribution is neglected here since it is observed at frequencies lower than the optical frequency.

For $\nu_m > \nu_c$ (the so-called fast cooling regime, expected at early times), the possibilities (marked A–C) are

$$F_\nu = F_{\nu_{\max}} \times \begin{cases} \nu_c^{-1/3}\nu^{1/3} & \nu < \nu_c < \nu_m \quad A \\ \nu_c^{1/2}\nu^{-1/2} & \nu_c < \nu < \nu_m \quad B \\ \nu_m^{(p-1)/2}\nu_c^{1/2}\nu^{-p/2} & \nu_c < \nu_m < \nu \quad C \end{cases} \tag{4}$$

At later times, $\nu_m < \nu_c$ and the plasma enters the slow cooling regime, in which (regions D–F)

$$F_\nu = F_{\nu_{\max}} \times \begin{cases} \nu_m^{-1/3}\nu^{1/3} & \nu < \nu_m < \nu_c \quad D \\ \nu_m^{(p-1)/2}\nu^{-(p-1)/2} & \nu_m < \nu < \nu_c \quad E \\ \nu_m^{(p-1)/2}\nu_c^{1/2}\nu^{-p/2} & \nu_m < \nu_c < \nu \quad F \end{cases} \tag{5}$$

Here, $\nu_m$ is the typical emission frequency from electrons at the peak of the distribution, namely having Lorentz factor $\gamma_m$, and is given by (for an on-axis observer)

$$\nu_m^{ob} = \frac{3}{4\pi}\frac{qB}{m_e c}\gamma_m^2\frac{\Gamma}{(1+z)} = 4.196 \times 10^6 B\gamma_m^2\frac{\Gamma}{(1+z)}\,\text{Hz}, \tag{6}$$

where $q$ is the electron's charge, $m_e$ is the electron's mass, $c$ is the speed of light, $B$ is the magnetic field, $\Gamma$ is the bulk motion Lorentz factor and $z$ is the redshift. The cooling frequency $\nu_c$ is the frequency of emission from electrons whose radiative cooling time is equal to the dynamical time,

$$\nu_c^{ob} = \frac{3}{4\pi}\frac{q}{m_e c}\left(\frac{6\pi m_e c^2}{\sigma_T}\right)^2\frac{\Gamma^3}{B^3 r^2(1+z)} = 2.26 \times 10^{45}\frac{\Gamma^3}{B^3 r^2(1+z)}\,\text{Hz}. \tag{7}$$

Here, $\sigma_T$ is Thomson's cross section, and $r$ is the plasma radius, which is related to the dynamical time (in the comoving frame) by $r \sim \Gamma c t_{\text{dyn}}$[12]. The peak flux is estimated by $F^{ob}_{\nu,peak} = \frac{1}{4\pi d_L^2}N_e P^{ob}_{\nu,\max}$, where $d_L$ is the luminosity distance, $N_e$ is the number of radiating particles and $P^{ob}_{\nu,\max} = P^{ob}_{tot}/\nu^{ob}_{peak}$, where $P^{ob}_{tot}$ is the total power radiated by synchrotron emission by a single electron at $\gamma_m$, and $\nu^{ob}_{peak} = \nu^{ob}_m$. This gives

$$P^{ob}_{\nu,max} = \frac{P^{ob}_{tot}}{\nu^{ob}_m} = \frac{\frac{4}{3}c\sigma_T\gamma_m^2\frac{B^2}{8\pi}\Gamma^2}{\frac{3}{4\pi}\frac{qB}{m_e c}\gamma_m^2\frac{\Gamma}{(1+z)}} = \frac{2}{9}\frac{m_e c^2\sigma_T}{q}B\Gamma(1+z) = 2.53 \times 10^{-22}B\Gamma(1+z)\ \text{erg s}^{-1}\,\text{Hz}^{-1} \tag{8}$$

[12]For simplicity, we neglect a possible factor of the order unity, as well as contribution form inverse-Compton (IC) cooling, which is negligible for the parameters used in this work.

We assume that all the ambient particles collected by the forward shock wave radiate. By assumption, the mass density is given by $\rho(r) = A/r^2$, implying that

$$N_e(r) = \frac{4\pi}{m_p}\int_0^r \rho(r')r'^2 dr' = \frac{4\pi A r}{m_p} \tag{9}$$

The proportionality constant $A$ is calculated assuming that prior to its final explosion, the progenitor star ejects mass at a constant rate and at a constant velocity, resulting in $\rho(r) = n(r)m_p = \frac{\dot{M}}{4\pi v_w r^2} \equiv Ar^{-2}$. For a Wolf-Rayet progenitor, the typical values are[41], $\dot{M} = 10^{-5} M_\odot \, \mathrm{yr}^{-1}$, and wind velocity $v_w = 10^8 \, \mathrm{cm\,s^{-1}}$. These values lead to $A = 5\times 10^{11} A_\star \, \mathrm{gr\ cm^{-1}}$.

***Supplementary Method 2a. Dynamics, magnetic field and electron's energy***

Following an initial acceleration phase, the plasma coasts at a nearly steady Lorentz factor $\Gamma_i$. Once it collects sufficient material from the ambient medium, $m_{ISM} \gtrsim m_i/\Gamma_i$ where $m_i$ is the initial ejected mass, the flow becomes similar, and its evolution is described by the well-known self-similar solution[42]. For an instantaneous explosion releasing energy $E$ that occurs into a density gradient this solution reads

$$\Gamma(E;r) = \left(\frac{9E}{16\pi\rho(r)c^2r^3}\right)^{1/2} = \left(\frac{9E}{16\pi A c^2 r}\right)^{1/2}. \tag{10}$$

The relation between the Lorentz factor, radius and observed time in this case was calculated by Ref.[43], $t^{\rm ob} \simeq (1+z)r/(2\Gamma^2(r)c)$, enabling to express the Lorentz factor as a function of the observed time during this phase,

$$\Gamma(E,A;t^{\rm obs.}) = \left(\frac{9E(1+z)}{32\pi A c^3 t^{\rm ob}}\right)^{1/4}. \tag{11}$$

Transition from the initial (coasting) to the later (self-similar expansion) phases occurs once $\Gamma(E,A;t^{\rm ob}) < \Gamma_i$[13]. Thus,

$$T_a = (1+z)\frac{9E}{32\pi A c^3 \Gamma_i^4}. \tag{12}$$

It is explicitly assumed that both the generation of magnetic field and acceleration of particles to high energies occur at the shock wave. The energy density behind the shock is given by the shock jump conditions, $u \simeq 4\Gamma^2 n m_p c^2$ where $m_p$ is the proton mass and $n$ is the particle number density in the surrounding medium. Therefore, during the initial (coasting) phase where $\Gamma = \Gamma_i$, this energy density is $u(t^{\rm obs.})_{\rm coasting} = (1+z)^2 A/(\Gamma_i^2 t_{\rm obs.}^2)$. We adopt the standard assumption that a fraction $\varepsilon_B$ of this energy is used in generating a magnetic field, and a fraction $\varepsilon_e$ is used in heating (accelerating) the electrons. During the initial (coasting) phase, this gives a magnetic field strength $B_{\rm initial} = (8\pi\varepsilon_B u)^{1/2} = 12\ (1+z)\ A_\star^{1/2}\Gamma_{i,1.5}^{-1} t_3^{\rm obs.\,-1}\ \varepsilon_{B,-2}^{1/2}$ G and typical electron Lorentz factor, $\gamma_{m,\rm initial} = \varepsilon_e \Gamma_i (m_p/m_e) = 5.5\times 10^3\ \Gamma_{i,1.5}\varepsilon_{e,-1}$. Here and below $Q_{,x} = Q/10^x$.

During the deceleration phase, a similar calculation with the use of Supplementary Equation (10) gives

$$B_{late} = \left(\frac{2048\pi^3A^3c^3(1+z)^3\varepsilon_B^2}{9E t^{\rm ob\,3}}\right)^{1/4} = 0.74\ \left(\frac{1+z}{2}\right)^{3/4} E_{53}^{-1/4} A_\star^{3/4} t_{\rm day}^{\rm ob\ -3/4}\ \varepsilon_{B,-2}^{1/2}\ \mathrm{G}, \tag{13}$$

[13] A second condition is that $t > t_{GRB}$, which is always met.

and

$$\gamma_{m,late} = \epsilon_e \Gamma \frac{m_p}{m_e} = \epsilon_e \left(\frac{m_p}{m_e}\right) \left(\frac{9E(1+z)}{32\pi A c^3 t^{\rm ob}}\right)^{1/4} = 2040 \left(\frac{1+z}{2}\right)^{1/4} E_{53}^{1/4} A_\star^{-1/4} t_{\rm day}^{{\rm ob}\ -1/4}\ \epsilon_{e,-1}. \quad (14)$$

***Supplementary Method 2b. Temporal and spectral signal***

Using these results in Supplementary Equations (6), (7) and (8) gives the parametric dependence of the key observed frequencies and flux:

$$\nu_m^{\rm obs.} = \begin{cases} 4.6 \times 10^{16}\, A_\star^{1/2} \Gamma_{i,1.5}^2 t_3^{\rm obs.\ -1}\ \epsilon_{e,-1}^2\ \epsilon_{B,-2}^{1/2}\ {\rm Hz} & ({\rm coasting}), \\ 7.2 \times 10^{13}\ \left(\frac{1+z}{2}\right)^{1/2} E_{53}^{1/2} t_{\rm day}^{{\rm ob}\ -3/2} \epsilon_{e,-1}^2 \epsilon_{B,-2}^{1/2}\ {\rm Hz} & ({\rm decay}), \end{cases} \quad (15)$$

$$\nu_c^{\rm obs.} = \begin{cases} 3 \times 10^{12} \left(\frac{1+z}{2}\right)^{-2}\ A_\star^{-3/2} \Gamma_{i,1.5}^2 t_3^{\rm obs.}\ \epsilon_{B,-2}^{-3/2}\ {\rm Hz} & ({\rm coasting}), \\ 3.7 \times 10^{13}\ \left(\frac{1+z}{2}\right)^{-3/2} E_{53}^{1/2} t_{\rm day}^{{\rm ob}\ 1/2} \epsilon_{B,-2}^{-3/2} A_\star^{-2}\ {\rm Hz} & ({\rm decay}), \end{cases} \quad (16)$$

and

$$F_{\nu,peak}^{\rm obs.} = \begin{cases} 6.1 \times 10^{-24} \left(\frac{1+z}{2}\right) d_{L,28.3}^{-2}\ A_\star^{3/2} \Gamma_{i,1.5}^2 \epsilon_{B,-2}^{1/2}\ {\rm erg\ cm^{-2}\ s^{-1}\ Hz^{-1}} & ({\rm coasting}), \\ 9.9 \times 10^{-25}\ \left(\frac{1+z}{2}\right)^{3/2} d_{L,28.3}^{-2} E_{53}^{1/2} A_\star \epsilon_{B,-2}^{1/2} t_{\rm day}^{{\rm ob}\ -1/2} {\rm erg\ cm^{-2}\ s^{-1}\ Hz^{-1}} & ({\rm decay}). \end{cases} \quad (17)$$

The results of Supplementary Equations (15) and (16) imply that both at early (coasting) and later (self-similar decay) phases, the peak frequency decreases with observed time, while the cooling frequency increases with time. This have two important consequences on the spectral and temporal evolution of the observed signal: (i) At high enough frequencies, the transition always occurs from region F ($\nu_c < \nu$) to region E ($\nu < \nu_c$);[14] and (ii) the transition always occurs at lower frequencies first- it will occur in the optical band before the X-ray band, regardless of whether the flow is in the coasting or in the decaying phase.

Using these results in Supplementary Equations (4) and (5) give the temporal and spectral dependence in each of the 6 possible regimes considered, both during the coasting and during the self-similar decay phases (see for consistency with Ref.[44]). These are summarized in Supplementary Table 7 and Supplementary Fig. 17.

These results can now be used to explain the observed signal, which is in an excellent agreement with the theoretical model. Provided that the transition to region E occurs after the end of the coasting phase, the light curve during the coasting phase is $\approx$ flat, $F_\nu \propto t_{\rm obs.}^{(2-p)/2} \sim t_{\rm obs.}^{0.0..-0.2}$, which is similar to the observed signal in the X-ray band in classes I and II. Since the transition to region E occurs earlier at longer wavelength, in some of the bursts in class I the optical band is already in region E, in which case a decay in the optical light curve, $F_\nu \propto t_{\rm obs.}^{(1-p)/2} \sim t_{\rm obs.}^{-0.5..-0.7}$ is expected. The only other option is that the transition to region E occurs at earlier times, in which case both the X-ray and the optical light curves show a decay, which is the relevant scenario for class III here.

Once the flow shifts to the self-similar decay phase, both the X-ray and the optical light curves decay as $F_\nu \propto t_{\rm obs.}^{(2-3p)/4} \sim t_{\rm obs.}^{-1.0..-1.3}$ if in region F, or alternatively $F_\nu \propto t_{\rm obs.}^{(1-3p)/4} \sim t_{\rm obs.}^{-1.25..-1.55}$ if in region E. However, the difference in the temporal index in between these two cases is only 1/4, and may not be easily identified due to the noise.

[14] At lower frequencies, it occurs from region D to region E

### *Supplementary Method 2c. Determining the physical properties of the outflow*

A flat X-ray plateau indicates that the flux in the X-ray band is in region F. Assuming, for simplicity a power-law index $p = 2$, Supplementary Equation (5) gives

$$\nu F_\nu(\mathrm{X})^{\mathrm{obs.}} = F^{\mathrm{obs.}}_{\nu,\mathrm{peak}} \nu_c^{1/2} \nu_m^{1/2} = \frac{1}{d_L^2} c^3 A \Gamma_i^4 \varepsilon_e \tag{18}$$

where we made use of Supplementary Equations (15), (16) and (17). Combining this result with Supplementary Equation (12), one finds that for bursts with known redshift, the transition time and X-ray flux provide a direct measurement of $\varepsilon_e$:

$$\varepsilon_e = \frac{d_L^2}{(1+z)} \frac{32\pi T_a}{9E} \nu F_\nu(\mathrm{X})^{\mathrm{obs.}} \tag{19}$$

Furthermore, the transition time provides a strong constraint on the ambient medium and the initial Lorentz factor. This is done by writing Supplementary Equation (12)

$$A\Gamma_i^4 = (1+z) \frac{9E}{32\pi c^3 T_a}, \tag{20}$$

which is a very robust result.

A further constrain can be put by using the temporal behaviour of the optical data. In class I, the optical light curve decays, namely the optical band is in region E, and $\nu F_\nu(\mathrm{U})^{\mathrm{obs.}} = \mathrm{F}^{\mathrm{obs.}}_{\nu,\mathrm{peak}} \nu_{\mathrm{m}}^{1/2} \nu_{\mathrm{U}}^{1/2}$ (for $p = 2$). Using Supplementary Equations (15), (17), (19) and using an observed optical band $\nu_{\mathrm{U}} = 8.65 \times 10^{14}$ Hz as well as fiducial values for $z = 1$, $E = 3.16 \times 10^{52}$erg, $\nu F_\nu(\mathrm{X})^{\mathrm{obs.}} = 3.16 \times 10^{-12}$ erg cm$^{-2}$ s$^{-1}$ at $T_a = 3000$ s and $\nu F_\nu(U)^{\mathrm{obs.}} = 10^{-12}$ erg cm$^{-2}$ s$^{-1}$ at 1000 s, one finds

$$A_\star^{7/4} \Gamma^3_{i,1.5} \varepsilon^{3/4}_{B,-2} = 4 \times 10^{-3}. \tag{21}$$

Thus, for a given density parameter ($A_\star$), there is a corresponding magnetic parameter ($\varepsilon_B$). One may therefore use an external knowledge of $\varepsilon_B$ (e.g., an absolute upper limit of $\varepsilon_B = 1$) to completely determine the values of $A_\star$ and therefore of $\Gamma_i$. We further note that in this case, the ratio of fluxes,

$$R_I \equiv \frac{\nu F_\nu(\mathrm{X})}{\nu F_\nu(\mathrm{U})} = \left(\frac{\nu_c}{\nu_{\mathrm{U}}}\right)^{1/2} \left(\frac{\nu_{\mathrm{X}}}{\nu_{\mathrm{U}}}\right)^{(2-p)/2} \tag{22}$$

is expected in the range $10 \leq R \leq 300$ for power law indices in the range $1.8 \leq p \leq 2.4$.

In class II, the optical light curve is flat, namely it is in region F. Supplementary Equations (19) and (20) are valid, and thus a direct measurement of $\varepsilon_e$ and of $A\Gamma_i^4$ exist. However, as $\nu_c < \nu_{\mathrm{U}}$, the ratio of the optical and X-ray fluxes is constant,

$$R_{II} \equiv \frac{\nu F_\nu(\mathrm{X})}{\nu F_\nu(\mathrm{U})} = \left(\frac{\nu_{\mathrm{X}}}{\nu_{\mathrm{U}}}\right)^{(2-p)/2} \approx 1 \tag{23}$$

Instead, we use the fact that $\nu_c$ increases with time (Supplementary Equation (16)) to argue that in this case at the transition time (Supplementary Equation (12)), $\nu > \nu_c$. This gives a lower limit on the magnetization,

$$\varepsilon^{3/2}_{B,-2} > 3.1 \times 10^{-3} \left(\frac{1+z}{2}\right)^{-1} E_{52.5} A_\star^{-5/2} \Gamma^{-2}_{i,1.5}. \tag{24}$$

Thus, in this case, for a given $A_\star$ there is a minimum value of the magnetic field, which is inversely proportional to $A_\star$. Thus, again, one may use an external constraint (such as $\varepsilon_B < 1$) to constrain a minimum value of $A_\star$ which translates to a maximum value of $\Gamma_i$.

Finally, in class III both the X-ray and the optical light curves decay, namely both are in region E. In this case, Supplementary Equation (20) is valid, but not Supplementary Equation (19). It is therefore not possible to obtain a direct measure of $\varepsilon_e$ in this case. The best constraint can be put by using the requirement that at the beginning of the plateau, at $\sim 100$ s, the X-ray frequency is already below the cooling frequency, $\nu_X < \nu_c$. This translates into an upper limit,

$$\left(\frac{1+z}{2}\right) A_\star^{3/2} \Gamma_{i,1.5}^{-2} \varepsilon_{B,-2}^{3/2} < 2.5 \times 10^{-6} \qquad (25)$$

This provides an upper limit on the value of $\varepsilon_B$ for a given $A_\star$, which increases as $A_\star$ decrease. Using external constraint, e.g., $\varepsilon_B = 0.01$ is therefore useful in providing an upper limit on $A_\star$, and a lower limit on $\Gamma_i$. Combined with a measurement of the optical flux, this also gives a lower limit on the value of $\varepsilon_e$.

The ratio of the X-ray to optical fluxes in this case is intermediate,

$$R_{III} \equiv \frac{\nu F_\nu(\mathrm{X})}{\nu F_\nu(\mathrm{U})} = \left(\frac{\nu_\mathrm{X}}{\nu_\mathrm{U}}\right)^{(3-p)/2} \approx 10...150 \qquad (26)$$

depending on the value of $p$.

## Supplementary Discussion: Comparison with other models aimed at explaining the X-ray plateau

As discussed in the Introduction of the main manuscript, a plethora of models were proposed in the literature in attempts to explain the X-ray plateau. Here we discuss some of the most recent works, focusing on three topics: energy injection, reverse shock, and viewing angle.

Metzger et al. (2011) propose a millisecond protomagnetar model for GRBs[45]. In this magnetar model, the energy released by dipole radiation is predicted to be transferred to the surroundings via a magnetar wind. This wind is heated by neutrinos just after the launch of the supernova shock from a core collapse of a massive star. The outflow is collimated into a bipolar jet by its interaction with the progenitor star. As the magnetar cools, the wind becomes ultrarelativistic and Poynting flux dominated on a time-scale comparable to that required for the jet to clear a cavity through the star. Therefore, in this model the magnetic dissipation and shocks explain the prompt emission and the steep decay phase that follows. The late time flares and the X-ray plateau are thought to be powered by continuous dissipation of magnetic energy or by extraction of the residual rotational energy. Indeed, assuming a magnetic field and a spin period typical of a fast rotating neutron star, Rowlinson et al. (2014)[46] explain the correlation between luminosity and duration of the plateau phase within the $1\sigma$ uncertainties[8] in the framework of this model. A later paper by Rea et al. (2015)[47] shows that the magnetar model can be reconciled within the GRB emission of the plateau only if supermagnetars with high magnetic field strength are allowed.

In a successive paper of Stratta et al. (2018)[48] for the first time a non-ideal modelling of spindown magnetar is fitted to the afterglow data for samples of 40 long and 13 short GRBs with a well-defined plateau. The conclusion reached in that paper is that the data of both short and long GRBs can be explained within the magnetar model. However, the difference between the short and long bursts is that the long GRBs are characterized by a lower magnetic field and longer spin period compared to the short GRBs. The correlation between magnetic field and spin period follows the established physics of the spin-up line for accreting neutron star (NS) in galactic binary systems. The $B - P$ relation obtained with this sample of

53 GRBs matches spin-up line predictions for the magnetar model with mass accretion rates expected in the GRB prompt phase. The latter are $\sim 11-14$ orders of magnitude higher than those inferred for the galactic accreting NSs.

Matsumoto et al. 2020[49] show a link between extended emission seen in some short GRBs and the plateau phase. They assume a continuous energy injection from a central compact object, such as a magnetar, for the origin of both emission phases. Such an extended emission lasting up to $10^2-10^3$ s is also reported in several long GRBs[50,51]. In our study, when analyzing the full sample of 222 GRBs with known redshift and a plateau phase, we found that when such an extended emission exists, it affects the slopes of both the plateau and the following self-similar phases. The slopes for those bursts that show extended emission are steeper in both the plateau and self similar phases ($<-0.7$ and $<-1.2$ respectively) than the limit we take as an indication for a plateau (temporal slope of $>-0.7$). These are therefore excluded from our final sample.

Zhao et al. 2020[52] argued that in a few cases, there is a steep decay following the plateau phase, after which a second plateau may exist. They interpreted this as an outcome of a central engine consisting of a rapidly spinning magnetar that collapses to a newborn black hole. When analyzing the 3 GRBs in their sample, we found that the evidence for a steep decay is not strong: the X-ray LC of these 3 bursts either contain flares, or very few data points exist.

In contrast to previous works, Çıkıntoğlu et al. (2020)[53] argue that a clear plateau phase may not be realized if the magnetic field of the nascent magnetar is in a transient rapid decay stage. Due to this stage, the spin-down power may decline too fast and cause a lack of the plateau phase. With this idea, they analyze the X-ray light curve of 6 GRBs without plateau and conclude that these GRBs might be hosting millisecond magnetars.

A different type of model was proposed by Lyutikov et al. (2017)[54] and Barkov et al. (2021)[55], in which the plateau phase, flares and possible steep slopes immediately after the plateau phase, are all explained by a dominant reverse shock. This reverse shock is different than the classical reverse shock [15] predicted as part of the fireball model[57,58] because it is assumed to propagate through ultrarelativistic, highly magnetized pulsar-like winds produced by long-lasting central engines. While this is an interesting idea, similar to the models discussed above, only very few GRBs observed until now seem to fit the predictions of this model. Moreover, those GRBs have several flares during their X-ray data which makes it difficult to draw a firm conclusion. Within the realm of the classical fireball model, contribution from the reverse shock is expected during the transition from the coasting to the similar phase. Indeed when analyzing the light curves of the GRBs in our sample we did see some evidence for a possible contribution from a reverse shock in the optical light curve (especially in GRB 110213A). However, a full analysis of this effect is beyond the scope of this manuscript, and will be presented elsewhere.

Steep slopes ($<-3$) immediately after the plateau phase (in this case called internal plateau) were first identified by Troja et al. 2007[59] in the X-ray light curve of GRB 070110. The analysis was done by fitting a flat X-ray light-curve up to  16000 s, followed by a sharp decline. However, when looking at the data, one can find that the flux between 8000s and 16000s may be due to a flare, implying that the data is consistent with a single slope (no plateau phase) and a large late time flare. If this interpretation is correct, there is a single decay slope, which is naturally much shallower than the one found by Troja et al. (2007)[59]. Recently, Tang et al. (2019)[33] increased the sample to 10 GRBs showing an internal plateau. A close look into this sample reveals that one can interpret the X-ray afterglow light-curve of those GRBs in more than one way. The first point is the effect of flares on the identification of the plateau, as well

[15]Emission from such a reverse shock can be easily dominated by that of the external forward shock in the X-ray band. In fact, the difference in magnitude between the two components is expected to be at least one order of magnitude[56].

as on the light curve decline. In the analysis carried by Tang et al. (2019)[33], no flares were assumed to exist in the X-ray light curve of those GRBs. However, when including the effects of flares, the temporal decline index changes. This is further enhanced by the fact that in some bursts the data is very sparse – in a few cases, in fact no data exists during the plateau at all, and the existence of a plateau is inferred only by the brightness of the flare (e.g., see the flare identification in the X-ray light curve of GRB 050730 by Chincarini at al. (2007)[60]).

Alternatively, internal plateaus might have a different origin - they may be powered by a long-lasting central engine, in a similar way to the X-ray flares, that might be powered by an extended central engine activity[61]. Such a model is commonly used to explain the internal plateau seen in the *Swift*-BAT or *Swift*-XRT light curve of short GRBs around a few hundred seconds ($\sim 180$ s)[62].

Clearly, once the effects of flares are isolated, the conclusions of the two analysis are different. Thus, overall, we conclude that the "internal plateaus" found in the X-ray light curve of some long GRBs are a matter of interpretation, and more data / analysis is needed. We thus suggest here a different interpretation, which is not inconsistent with the data.

Another suggestion, recently promoted by Oganesyan et al. (2020)[63] and Beniamini et al. (2020)[64] is that of a structured jet viewed off-axis. These works were motivated by the observation of an off-axis jet detected from the very low luminosity GRB 170817A, associated to GW 170817, the first gravitational waves associated to the merger of two neutron stars. While Oganesyan et al. (2020)[63] explained the plateau phase by high-latitude emission, Beniamini et al. (2020)[64] argued in favour of a structured jet on the near-core lines of sight. The addition of degrees of freedom in these models, might enable good explanation to bursts which show a plateau.

In this work, we adopted and developed a much simpler model than all of the above. This model does not require any addition or modification to the classical GRB fireball model. Instead, we simply consider a different region of parameter space: a flow having an initial Lorentz factor of the order of few tens, propagating into a wind environment, as proposed earlier by Shen & Matzner (2012)[65]. We find that this model can naturally explain the observed plateau, both in the optical and X-rays, within the framework of synchrotron emission from particles accelerated to a power-law distribution by the forward shock wave. We further find that these assumptions lead to a typical ambient density of up to 2 orders of magnitude below the expectation from a wind produced by a Wolf-Rayet star. A similar conclusion with different approach is drawn by Chrimes et al. (2022)[66] that the different progenitors with weaker winds or very dense ISM environments are needed to confine the termination shock radius defined at transition from the wind to ISM medium.

In our work, we show that this simple theoretical idea matches many observations obtained in both X-ray and optical bands. We further extended the theory to show how it can be used to extract meaningful information on the outflow properties, and showed that there is no contradiction between the parameter values required by our model and those known from GRBs which do not show a plateau.

## Supplementary References


**1.** Srinivasaragavan, G. P. *et al.* On the Investigation of the Closure Relations for Gamma-Ray Bursts Observed by Swift in the Post-plateau Phase and the GRB Fundamental Plane. *Astrophys. J.* **903**, 18, DOI: 10.3847/1538-4357/abb702 (2020). 2009.06740.

**2.** Dainotti, M. G. *et al.* The Optical Luminosity-Time Correlation for More than 100 Gamma-Ray Burst Afterglows. *Astrophys. J.* **905**, L26, DOI: 10.3847/2041-8213/abcda9 (2020). 2011.14493.

3. Dainotti, M. G. *et al.* The X-Ray Fundamental Plane of the Platinum Sample, the Kilonovae, and the SNe Ib/c Associated with GRBs. *The Astrophysical Journal* **904**, 97, DOI: 10.3847/1538-4357/abbe8a (2020).
4. Dainotti, M. G. *et al.* Closure relations during the plateau emission of Swift GRBs and the fundamental plane. *Publications of the Astronomical Society of Japan* **73**, 970–1000, DOI: 10.1093/pasj/psab057 (2021).
5. Dainotti, M. G. *et al.* On the Existence of the Plateau Emission in High-energy Gamma-Ray Burst Light Curves Observed by Fermi-LAT. *The Astrophysical Journal Supplement Series* **255**, 13, DOI: 10.3847/1538-4365/abfe17 (2021).
6. Gehrels, N. *et al.* The Swift Gamma-Ray Burst Mission. *Astrophys. J.* **611**, 1005–1020, DOI: 10.1086/422091 (2004). astro-ph/0405233.
7. Dainotti, M. G., Postnikov, S., Hernandez, X. & Ostrowski, M. A Fundamental Plane for Long Gamma-Ray Bursts with X-Ray Plateaus. *The Astrophysical Journal* **825**, L20, DOI: 10.3847/2041-8205/825/2/l20 (2016).
8. Dainotti, M. G. *et al.* A Study of the Gamma-Ray Burst Fundamental Plane. *The Astrophysical Journal* **848**, 88, DOI: 10.3847/1538-4357/aa8a6b (2017).
9. Willingale, R. *et al.* Testing the Standard Fireball Model of Gamma-Ray Bursts Using Late X-Ray Afterglows Measured by Swift. *Astrophys. J.* **662**, 1093–1110, DOI: 10.1086/517989 (2007). astro-ph/0612031.
10. Zhang, B. *et al.* Physical Processes Shaping Gamma-Ray Burst X-Ray Afterglow Light Curves: Theoretical Implications from the Swift X-Ray Telescope Observations. *Astrophys. J.* **642**, 354–370, DOI: 10.1086/500723 (2006). astro-ph/0508321.
11. Ioka, K., Kobayashi, S. & Zhang, B. Variabilities of Gamma-Ray Burst Afterglows: Long-acting Engine, Anisotropic Jet, or Many Fluctuating Regions? *Astrophys. J.* **631**, 429–434, DOI: 10.1086/432567 (2005). astro-ph/0409376.
12. Boër, M. & Gendre, B. Evidences for two Gamma-Ray Burst afterglow emission regimes. *Astron. Astrophys.* **361**, L21–L24 (2000). astro-ph/0008385.
13. Gendre, B., Galli, A. & Boër, M. X-Ray Afterglow Light Curves: Toward A Standard Candle? *Astrophys. J.* **683**, 620–629, DOI: 10.1086/589805 (2008).
14. Oates, S. R. *et al.* A correlation between the intrinsic brightness and average decay rate of Swift/UVOT gamma-ray burst optical/ultraviolet light curves. *Mon. Not. R. Astron. Soc.* **426**, L86–L90, DOI: 10.1111/j.1745-3933.2012.01331.x (2012). 1208.1856.
15. Racusin, J. L., Oates, S. R., de Pasquale, M. & Kocevski, D. A Correlation between the Intrinsic Brightness and Average Decay Rate of Gamma-Ray Burst X-Ray Afterglow Light Curves. *Astrophys. J.* **826**, 45, DOI: 10.3847/0004-637X/826/1/45 (2016). 1605.00719.
16. Dereli, H. *et al.* A Study of GRBs with Low-luminosity Afterglows. *Astrophys. J.* **850**, 117, DOI: 10.3847/1538-4357/aa947d (2017).
17. Lazzati, D. & Perna, R. X-ray flares and the duration of engine activity in gamma-ray bursts. *Mon. Not. R. Astron. Soc.* **375**, L46–L50, DOI: 10.1111/j.1745-3933.2006.00273.x (2007). astro-ph/0610730.

**18.** Evans, P. A. *et al.* An online repository of Swift/XRT light curves of $\gamma$-ray bursts. *Astron. Astrophys.* **469**, 379–385, DOI: 10.1051/0004-6361:20077530 (2007). 0704.0128.

**19.** Evans, P. A. *et al.* Methods and results of an automatic analysis of a complete sample of Swift-XRT observations of GRBs. *Mon. Not. R. Astron. Soc.* **397**, 1177–1201, DOI: 10.1111/j.1365-2966.2009.14913.x (2009). 0812.3662.

**20.** Goodman, J. & Weare, J. Ensemble samplers with affine invariance. *Communications in Applied Mathematics and Computational Science* **5**, 65–80, DOI: 10.2140/camcos.2010.5.65 (2010).

**21.** Zaninoni, E., Bernardini, M. G., Margutti, R., Oates, S. & Chincarini, G. Gamma-ray burst optical light-curve zoo: comparison with X-ray observations. *Astron. Astrophys.* **557**, A12, DOI: 10.1051/0004-6361/201321221 (2013). 1303.6924.

**22.** De Pasquale, M. *et al.* The central engine of GRB 130831A and the energy breakdown of a relativistic explosion. *Mon. Not. R. Astron. Soc.* **455**, 1027–1042, DOI: 10.1093/mnras/stv2280 (2016). 1509.09234.

**23.** Filgas, R. *et al.* GRB 091029: at the limit of the fireball scenario. *Astron. Astrophys.* **546**, A101, DOI: 10.1051/0004-6361/201219583 (2012). 1209.4658.

**24.** Cucchiara, A. *et al.* Constraining Gamma-Ray Burst Emission Physics with Extensive Early-time, Multiband Follow-up. *Astrophys. J.* **743**, 154, DOI: 10.1088/0004-637X/743/2/154 (2011). 1107.3352.

**25.** de Pasquale, M. *et al.* VizieR Online Data Catalog: Photometry of the afterglow of GRB 130831A (De Pasquale+, 2016). *VizieR Online Data Catalog* J/MNRAS/455/1027 (2018).

**26.** Izzo, L. *et al.* Signatures of a jet cocoon in early spectra of a supernova associated with a $\gamma$-ray burst. *Nature* **565**, 324–327, DOI: 10.1038/s41586-018-0826-3 (2019). 1901.05500.

**27.** Racusin, J. L. *et al.* Fermi and Swift Gamma-ray Burst Afterglow Population Studies. *Astrophys. J.* **738**, 138, DOI: 10.1088/0004-637X/738/2/138 (2011). 1106.2469.

**28.** Oates, S. R. *et al.* A statistical study of gamma-ray burst afterglows measured by the Swift Ultraviolet Optical Telescope. *Mon. Not. R. Astron. Soc.* **395**, 490–503, DOI: 10.1111/j.1365-2966.2009.14544.x (2009). 0901.3597.

**29.** Zafar, T. *et al.* The extinction curves of star-forming regions from z = 0.1 to 6.7 using GRB afterglow spectroscopy. *Astron. Astrophys.* **532**, A143, DOI: 10.1051/0004-6361/201116663 (2011). 1102.1469.

**30.** Schlegel, D. J., Finkbeiner, D. P. & Davis, M. Maps of Dust Infrared Emission for Use in Estimation of Reddening and Cosmic Microwave Background Radiation Foregrounds. *Astrophys. J.* **500**, 525–553, DOI: 10.1086/305772 (1998). astro-ph/9710327.

**31.** Schady, P. *et al.* Dust and metal column densities in gamma-ray burst host galaxies. *Mon. Not. R. Astron. Soc.* **401**, 2773–2792, DOI: 10.1111/j.1365-2966.2009.15861.x (2010). 0910.2590.

**32.** Pei, Y. C. Interstellar Dust from the Milky Way to the Magellanic Clouds. *Astrophys. J.* **395**, 130, DOI: 10.1086/171637 (1992).

**33.** Tang, C.-H., Huang, Y.-F., Geng, J.-J. & Zhang, Z.-B. Statistical Study of Gamma-Ray Bursts with a Plateau Phase in the X-Ray Afterglow. *Astrophys. J.* **245**, 1, DOI: 10.3847/1538-4365/ab4711 (2019). 1905.07929.

**34.** Dainotti, M. G., Ostrowski, M. & Willingale, R. Towards a standard gamma-ray burst: tight correlations between the prompt and the afterglow plateau phase emission. *Monthly Notices of the Royal Astronomical Society* **418**, 2202–2206, DOI: 10.1111/j.1365-2966.2011.19433.x (2011). https://academic.oup.com/mnras/article-pdf/418/4/2202/18747557/mnras0418-2202.pdf.

**35.** Dainotti, M. *et al.* Luminosity–time and luminosity–luminosity correlations for GRB prompt and afterglow plateau emissions. *Monthly Notices of the Royal Astronomical Society* **451**, 3898–3908, DOI: 10.1093/mnras/stv1229 (2015). https://academic.oup.com/mnras/article-pdf/451/4/3898/3889902/stv1229.pdf.

**36.** Dainotti, M. *Gamma-ray Burst Correlations*. 2053-2563 (IOP Publishing, 2019).

**37.** Spitkovsky, A. On the Structure of Relativistic Collisionless Shocks in Electron-Ion Plasmas. *Astrophys. J.* **673**, L39–L42, DOI: 10.1086/527374 (2008). 0706.3126.

**38.** Wijers, R. A. M. J. & Galama, T. J. Physical Parameters of GRB 970508 and GRB 971214 from Their Afterglow Synchrotron Emission. *Astrophys. J.* **523**, 177–186, DOI: 10.1086/307705 (1999). astro-ph/9805341.

**39.** Meszaros, P. & Rees, M. J. Relativistic fireballs and their impact on external matter - Models for cosmological gamma-ray bursts. *Astrophys. J.* **405**, 278–284, DOI: 10.1086/172360 (1993).

**40.** Sari, R., Piran, T. & Narayan, R. Spectra and Light Curves of Gamma-Ray Burst Afterglows. *Astrophys. J.* **497**, L17+, DOI: 10.1086/311269 (1998). arXiv:astro-ph/9712005.

**41.** Chevalier, R. A. & Li, Z.-Y. Wind Interaction Models for Gamma-Ray Burst Afterglows: The Case for Two Types of Progenitors. *Astrophys. J.* **536**, 195–212, DOI: 10.1086/308914 (2000). astro-ph/9908272.

**42.** Blandford, R. D. & McKee, C. F. Fluid dynamics of relativistic blast waves. *Physics of Fluids* **19**, 1130–1138, DOI: 10.1063/1.861619 (1976).

**43.** Pe'er, A. & Wijers, R. A. M. J. The Signature of a Wind Reverse Shock in Gamma-Ray Burst Afterglows. *Astrophys. J.* **643**, 1036–1046, DOI: 10.1086/500969 (2006). arXiv:astro-ph/0511508.

**44.** Gao, H., Lei, W.-H., Zou, Y.-C., Wu, X.-F. & Zhang, B. A complete reference of the analytical synchrotron external shock models of gamma-ray bursts. *New Astronomy Review* **57**, 141–190, DOI: 10.1016/j.newar.2013.10.001 (2013). 1310.2181.

**45.** Metzger, B. D., Giannios, D., Thompson, T. A., Bucciantini, N. & Quataert, E. The protomagnetar model for gamma-ray bursts. *Mon. Not. R. Astron. Soc.* **413**, 2031–2056, DOI: 10.1111/j.1365-2966.2011.18280.x (2011). 1012.0001.

**46.** Rowlinson, A. *et al.* Constraining properties of GRB magnetar central engines using the observed plateau luminosity and duration correlation. *Monthly Notices of the Royal Astronomical Society* **443**, 1779–1787, DOI: 10.1093/mnras/stu1277 (2014). https://academic.oup.com/mnras/article-pdf/443/2/1779/3703047/stu1277.pdf.

**47.** Rea, N. *et al.* Constraining the GRB-magnetar model by means of the Galactic pulsar population. *The Astrophysical Journal* **813**, 92, DOI: 10.1088/0004-637x/813/2/92 (2015).

**48.** Stratta, G., Dainotti, M. G., Dall'Osso, S., Hernandez, X. & De Cesare, G. On the Magnetar Origin of the GRBs Presenting X-Ray Afterglow Plateaus. *Astrophys. J.* **869**, 155, DOI: 10.3847/1538-4357/aadd8f (2018). 1804.08652.

**49.** Matsumoto, T., Kimura, S. S., Murase, K. & Mészáros, P. Linking extended and plateau emissions of short gamma-ray bursts. *Mon. Not. R. Astron. Soc.* **493**, 783–791, DOI: 10.1093/mnras/staa305 (2020). 2001.09851.

**50.** Bostancı, Z. F., Kaneko, Y. & Göğüş, E. Gamma-ray bursts with extended emission observed with BATSE. *Mon. Not. R. Astron. Soc.* **428**, 1623–1630, DOI: 10.1093/mnras/sts157 (2013). 1210.2399.

**51.** Kaneko, Y., Bostancı, Z. F., Göğüş, E. & Lin, L. Short gamma-ray bursts with extended emission observed with Swift/BAT and Fermi/GBM. *Mon. Not. R. Astron. Soc.* **452**, 824–837, DOI: 10.1093/mnras/stv1286 (2015). 1506.05899.

**52.** Zhao, L. *et al.* The Second Plateau in X-Ray Afterglow Providing Additional Evidence for Rapidly Spinning Magnetars as the GRB Central Engine. *Astrophys. J.* **896**, 42, DOI: 10.3847/1538-4357/ab8f91 (2020). 2005.00768.

**53.** Çıkıntoğlu, S., Şaşmaz Muş, S. & Ekşi, K. Y. The initial evolution of millisecond magnetars: an analytical solution. *Mon. Not. R. Astron. Soc.* **496**, 2183–2190, DOI: 10.1093/mnras/staa1556 (2020). 1910.00554.

**54.** Lyutikov, M. & Camilo Jaramillo, J. Early GRB Afterglows from Reverse Shocks in Ultra-relativistic, Long-lasting Winds. *Astrophys. J.* **835**, 206, DOI: 10.3847/1538-4357/835/2/206 (2017). 1612.01162.

**55.** Barkov, M. V., Luo, Y. & Lyutikov, M. Dynamics and Emission of Wind-powered Afterglows of Gamma-Ray Bursts: Flares, Plateaus, and Steep Decays. *Astrophys. J.* **907**, 109, DOI: 10.3847/1538-4357/abd5c2 (2021). 2004.13600.

**56.** Kumar, P. & Zhang, B. The physics of gamma-ray bursts and relativistic jets. *Phys. Rep.* **561**, 1–109, DOI: 10.1016/j.physrep.2014.09.008 (2015). 1410.0679.

**57.** Uhm, Z. L. & Beloborodov, A. M. On the Mechanism of Gamma-Ray Burst Afterglows. *Astrophys. J.* **665**, L93–L96, DOI: 10.1086/519837 (2007). astro-ph/0701205.

**58.** Genet, F., Daigne, F. & Mochkovitch, R. Can the early X-ray afterglow of gamma-ray bursts be explained by a contribution from the reverse shock? *Mon. Not. R. Astron. Soc.* **381**, 732–740, DOI: 10.1111/j.1365-2966.2007.12243.x (2007). astro-ph/0701204.

**59.** Troja, E. *et al.* Swift Observations of GRB 070110: An Extraordinary X-Ray Afterglow Powered by the Central Engine. *Astrophys. J.* **665**, 599–607, DOI: 10.1086/519450 (2007). astro-ph/0702220.

**60.** Chincarini, G. *et al.* The First Survey of X-Ray Flares from Gamma-Ray Bursts Observed by Swift: Temporal Properties and Morphology. *Astrophys. J.* **671**, 1903–1920, DOI: 10.1086/521591 (2007). astro-ph/0702371.

**61.** Ghisellini, G., Ghirlanda, G., Nava, L. & Firmani, C. "Late Prompt" Emission in Gamma-Ray Bursts? *Astrophys. J.* **658**, L75–L78, DOI: 10.1086/515570 (2007). astro-ph/0701430.

**62.** Lü, H.-J., Zhang, B., Lei, W.-H., Li, Y. & Lasky, P. D. The Millisecond Magnetar Central Engine in Short GRBs. *Astrophys. J.* **805**, 89, DOI: 10.1088/0004-637X/805/2/89 (2015). 1501.02589.

**63.** Oganesyan, G. *et al.* Structured Jets and X-Ray Plateaus in Gamma-Ray Burst Phenomena. *Astrophys. J.* **893**, 88, DOI: 10.3847/1538-4357/ab8221 (2020). 1904.08786.

**64.** Beniamini, P., Duque, R., Daigne, F. & Mochkovitch, R. X-ray plateaus in gamma-ray bursts' light curves from jets viewed slightly off-axis. *Mon. Not. R. Astron. Soc.* **492**, 2847–2857, DOI: 10.1093/mnras/staa070 (2020). 1907.05899.

**65.** Shen, R. & Matzner, C. D. Coasting External Shock in Wind Medium: An Origin for the X-Ray Plateau Decay Component in Swift Gamma-Ray Burst Afterglows. *Astrophys. J.* **744**, 36, DOI: 10.1088/0004-637X/744/1/36 (2012). 1109.3453.

**66.** Chrimes, A. A. *et al.* Towards an understanding of long gamma-ray burst environments through circumstellar medium population synthesis predictions. *Mon. Not. R. Astron. Soc.* DOI: 10.1093/mnras/stac1796 (2022). 2206.13595.

| | GRB names | 080607 | 091029 | 110213A | 130831A |
|---|---|---|---|---|---|
| X-rays | $\log(T_{a,X}/s)$ | $3.35^{+0.06}_{-0.05}$ | $4.15^{+0.05}_{-0.10}$ | $3.13^{+0.05}_{-0.06}$ | $2.87^{+0.05}_{-0.04}$ |
| | $log(T_{b,X}/s)$ | $4.07^{+0.05}_{-0.04}$ | ... | $3.97^{+0.03}_{-0.03}$ | ... |
| | $\alpha_{p,X}$ | $0.08^{+0.23}_{-0.23}$ | $-0.22^{+0.09}_{-0.06}$ | $0.30^{+0.14}_{-0.11}$ | $0.04^{+0.19}_{-0.18}$ |
| | $\alpha_{A1,X}$ | $-2.24^{+0.11}_{-0.12}$ | $-1.14^{+0.03}_{-0.03}$ | $-1.06^{+0.05}_{-0.05}$ | $-1.07^{+0.03}_{-0.04}$ |
| | $\alpha_{A2,X}$ | $-1.44^{+0.06}_{-0.06}$ | ... | $-1.94^{+0.04}_{-0.03}$ | ... |
| | $\beta_{p,X}$ | $0.82^{+0.15}_{-0.15}$ | $1.06^{+0.12}_{-0.12}$ | $0.98^{+0.11}_{-0.10}$ | $0.83^{+0.14}_{-0.11}$ |
| | $\beta_{A1,X}$ | $1.20^{+0.16}_{-0.15}$ | $1.05^{+0.12}_{-0.12}$ | $1.02^{+0.06}_{-0.05}$ | $0.72^{+0.12}_{-0.11}$ |
| | $\beta_{A2,X}$ | $1.13^{+0.20}_{-0.19}$ | ... | $1.14^{+0.21}_{-0.19}$ | ... |
| Optical | $\log(T_{a,O}/s)$ | $3.40^{+0.05}_{-0.02}$ | $4.19^{+0.03}_{-0.03}$ | $3.33^{+0.003}_{-0.01}$ | $3.69^{+0.01}_{-0.02}$ |
| | $\log(T_{b,O}/s)$ | ... | ... | $3.71^{+0.01}_{-0.01}$ | 931.0 |
| | $\alpha_{p,O}$ | $-0.49^{+0.01}_{-0.02}$ | $-0.36^{+0.01}_{-0.02}$ | $-0.61^{+0.02}_{-0.02}$ | $-0.4^{+0.01}_{-0.01}$ |
| | $\alpha_{A1,O}$ | $-1.22^{+0.02}_{-0.01}$ | $-1.06^{+0.01}_{-0.01}$ | $0.68^{+0.07}_{-0.07}$ | $-1.49^{+0.04}_{-0.03}$ |
| | $\alpha_{A2,O}$ | ... | ... | $-1.56^{+0.02}_{-0.02}$ | ... |
| | $\beta_{p,O}$ | $-0.84^{+0.01}_{-0.01}$ | $-0.46^{+0.06}_{-0.06}$ | ... | ... |
| | $\beta_{A1,O}$ | $-0.80^{0.0}_{0.0}$ | $-0.32^{+0.05}_{-0.06}$ | $-1.12^{+0.24}_{-0.24}$ | ... |
| | $\beta_{A2,O}$ | ... | $-0.34^{+0.06}_{-0.06}$ | $-1.22^{+0.18}_{-0.18}$ | ... |
| | References for optical spectral indices: | Ref.[21] | Ref.[23] | Ref.[24] | |

**Supplementary Table 1. Class I (flat X-ray plateau and decaying optical plateau).** The first column separate the X-rays and Optical parameters. Row 1: GRB names in this class. Row 2 and 3: temporal breaks in the X-ray light curves at the end of the plateau phase and later time respectively. Row 4 to 9: temporal and spectral slopes in the X-ray band. Row 10 and 11: temporal breaks in the optical light curves at the end of the plateau phase and at later time respectively. Row 12 to 17: temporal and spectral slopes in the optical band. The errors correspond to a significance of one sigma. See additional details in Supplementary Method 1a, X-ray data and fitting process as well as in Supplementary Method 1b, Optical data and fitting process.

| | GRB name | 060605 | 060614 | 060729 | 080310 | 100418A | 171205A |
|---|---|---|---|---|---|---|---|
| X-rays | $\log(T_{\rm a,X}/{\rm s})$ | $3.69^{+0.11}_{-0.16}$ | $4.53^{+0.03}_{-0.03}$ | $4.58^{+0.04}_{-0.02}$ | $4.04^{+0.04}_{-0.04}$ | $4.90^{+0.11}_{-0.07}$ | $4.96^{+0.04}_{-0.04}$ |
| | $\log(T_{\rm b,X}/{\rm s})$ | $4.18^{+0.09}_{-0.06}$ | $5.13^{+0.09}_{-0.09}$ | $5.15^{+0.04}_{-0.04}$ | ... | ... | ... |
| | $\alpha_{\rm p,X}$ | $-0.41^{+0.08}_{-0.04}$ | $0.02^{+0.05}_{-0.05}$ | $-0.14^{+0.02}_{-0.02}$ | $-0.21^{+0.10}_{-0.10}$ | $-0.09^{+0.06}_{-0.06}$ | $0.21^{+0.09}_{-0.09}$ |
| | $\alpha_{\rm A1,X}$ | $-1.24^{+0.15}_{-0.25}$ | $-1.41^{+0.13}_{-0.11}$ | $-0.87^{+0.05}_{-0.06}$ | $-1.55^{+0.06}_{-0.06}$ | $-1.39^{+0.09}_{-0.13}$ | $-1.06^{+0.05}_{-0.05}$ |
| | $\alpha_{\rm A2,X}$ | $-2.25^{+0.14}_{-0.17}$ | $-2.23^{+0.12}_{-0.15}$ | $-1.41^{+0.02}_{-0.02}$ | ... | ... | ... |
| | $\beta_{\rm p,X}$ | $0.90^{+0.14}_{-0.07}$ | $0.74^{+0.11}_{-0.05}$ | $0.97^{+0.04}_{-0.04}$ | $1.14^{+0.12}_{-0.07}$ | $0.9^{+0.4}_{-0.3}$ | $0.55^{+0.21}_{-0.10}$ |
| | $\beta_{\rm A1,X}$ | $1.16^{+0.17}_{-0.17}$ | $0.92^{+0.16}_{-0.15}$ | $0.98^{+0.04}_{-0.04}$ | $0.89^{+0.16}_{-0.15}$ | $0.84^{+0..32}_{-0.29}$ | $0.94^{+0.23}_{-0.22}$ |
| | $\beta_{\rm A2,X}$ | $1.08^{+0.20}_{-0.15}$ | ... | ... | ... | ... | ... |
| Optical | $\log(T_{\rm a,O}/{\rm s})$ | $2.77^{+0.02}_{-0.03}$ | $4.47^{+0.02}_{-0.04}$ | $4.78^{+0.05}_{-0.05}$ | $3.54^{+0.10}_{-0.12}$ | $4.67^{+0.10}_{-0.14}$ | $4.55^{+0.02}_{-0.04}$ |
| | $\log(T_{\rm b,O}/{\rm s})$ | $3.47^{+0.05}_{-0.02}$ | $4.84^{+0.05}_{-0.03}$ | ... | $4.60^{+0.27}_{-0.62}$ | ... | $5.25^{+0.04}_{-0.04}$ |
| | $\alpha_{\rm p,O}$ | $0.18^{+0.01}_{-0.02}$ | $0.10^{+0.04}_{-0.03}$ | $-0.07^{+0.03}_{-0.03}$ | $-0.05^{+0.08}_{-0.08}$ | $0.22^{+0.07}_{-0.08}$ | $0.15^{+0.01}_{-0.01}$ |
| | $\alpha_{\rm A1,O}$ | $-1.02^{+0.01}_{-0.02}$ | $-0.96^{+0.16}_{-0.15}$ | $-1.49^{+0.07}_{-0.08}$ | $-1.02^{+0.16}_{-0.12}$ | $-0.70^{+0.05}_{-0.05}$ | $-0.65^{+0.01}_{-0.01}$ |
| | $\alpha_{\rm A2,O}$ | $-1.62^{+0.02}_{-0.01}$ | $-2.02^{+0.08}_{-0.08}$ | ... | $-1.26^{+0.19}_{-0.33}$ | ... | $0.44^{+0.01}_{-0.01}$ |
| | $\beta_{\rm p1,O}$ | $-1.32^{+0.03}_{-0.03}$ | $-0.30^{0.0}_{0.0}$ | $-0.59^{0.0}_{0.0}$ | $-0.97^{+0.04}_{-0.04}$ | ... | $-0.65^{+0.39}_{-0.39}$ |
| | $\beta_{\rm p2,O}$ | ... | ... | $-0.88^{0.0}_{0.01}$ | ... | ... | $-0.83^{+0.19}_{-0.19}$ |
| | $\beta_{\rm A1,O}$ | $-1.14^{+0.02}_{-0.02}$ | $-0.35^{0.0}_{0.0}$ | $-0.54^{0.0}_{0.0}$ | $-0.88^{+0.04}_{-0.04}$ | $-1.19^{+0.02}_{-0.02}$ | $-0.77^{+0.15}_{-0.15}$ |
| | $\beta_{\rm A2,O}$ | ... | ... | ... | ... | ... | $-0.88^{+0.60}_{-0.60}$ |
| | References for optical spectral indices: | Ref.[21] | Ref.[21] | Ref.[21] | Ref.[21] | Ref.[21] | |

**Supplementary Table 2. Class II (both X-ray and optical plateaus are flat).** Columns and Rows are as defined in Supplementary Table 1.

| | GRB name | 050319 | 060714 | 061121 |
|---|---|---|---|---|
| X-rays | $\log(T_{\rm a,X}/{\rm s})$ | $4.50^{+0.06}_{-0.06}$ | $3.66^{+0.13}_{-0.10}$ | $3.96^{+0.03}_{-0.03}$ |
| | $\alpha_{\rm p,X}$ | $-0.55^{+0.02}_{-0.02}$ | $-0.42^{+0.10}_{-0.11}$ | $-0.59^{+0.02}_{-0.01}$ |
| | $\alpha_{\rm A1,X}$ | $-1.44^{+0.11}_{-0.12}$ | $-1.24^{+0.03}_{-0.04}$ | $-1.42^{+0.02}_{-0.02}$ |
| | $\beta_{\rm p,X}$ | $1.00^{+0.09}_{-0.06}$ | $0.88^{+0.15}_{-0.14}$ | $0.84^{+0.30}_{-0.28}$ |
| | $\beta_{\rm A1,X}$ | $1.18^{+0.21}_{-0.20}$ | $1.08^{+0.20}_{-0.19}$ | $0.85^{+0.07}_{-0.07}$ |
| | $\beta_{\rm A2,X}$ | ... | ... | $0.77^{+0.10}_{-0.10}$ |
| Optical | $\log(T_{\rm a,O}/{\rm s})$ | $4.55^{+0.04}_{-0.04}$ | $3.77^{+0.16}_{-0.18}$ | $4.14^{+0.35}_{-0.32}$ |
| | $\alpha_{\rm p,O}$ | $-0.59^{+0.01}_{-0.01}$ | $-0.27^{+0.09}_{-0.09}$ | $-0.48^{+0.05}_{-0.05}$ |
| | $\alpha_{\rm A1,O}$ | $-0.64^{+0.012}_{-0.011}$ | $-0.61^{+0.27}_{-0.28}$ | $-1.08^{+0.27}_{-0.64}$ |
| | $\beta_{\rm p,O}$ | $-0.36^{0.0}_{0.0}$ | $-0.44^{+0.04}_{-0.04}$ | $-0.68^{+0.06}_{-0.06}$ |
| | $\beta_{\rm A1,O}$ | $-0.61^{0.0}_{0.0}$ | $-0.98^{+0.11}_{-0.11}$ | $-0.68^{+0.02}_{-0.02}$ |
| | References for optical spectral indices: | Ref.[21] | Ref.[29] | Ref.[21] |

**Supplementary Table 3. Class III (both X-ray and optical plateaus are decaying).** Columns and Rows are as defined in Supplementary Table 1.

| $\log(T_a/s)$ | $\log(T_b/s)$ | N | $\alpha_p$ | $\alpha_{A1}$ | $\alpha_{A2}$ | $\log(V)$ |
|---|---|---|---|---|---|---|
| $4.53^{+0.03}_{-0.03}$ | $5.13^{+0.09}_{-0.09}$ | $-0.68^{+0.02}_{-0.02}$ | $0.02^{+0.05}_{-0.05}$ | $-1.41^{+0.13}_{-0.11}$ | $-2.23^{+0.12}_{-0.15}$ | $-11.54^{+2.34}_{-2.55}$ |

**Supplementary Table 4. X-ray temporal fit parameters of GRB 060614 with the Bayesian analysis tool *emcee*.** $T_a$ is the break time at the end of the plateau phase and $T_b$ is the jet break time, both are presented in a log scales. N is the normalization of the fit. The slopes $\alpha_p$, $\alpha_{A1}$, $\alpha_{A2}$ are obtained during the plateau phase, self-similar phase and after the jet break time respectively. V is the intrinsic scatter of the data presented in a log scale. The errors correspond to a significance of one sigma.

| GRB name | $A_{U,Gal.}$ | $A_{U,host}$ | Extinction law | note |
|---|---|---|---|---|
| 050319 | 0.052 | 0.53±0.012 | MW | (V-band)[21] |
| 060605 | 0.255 | 1.33±0.10 | MW | (CR-band)[21] |
| 060614 | 0.107 | 0.23±0.18 | MW | (U-band)[21] |
| 060714 | 0.378 | $1.53^{+0.65}_{-0.58}$ | ... | $Av_{host} = 0.79^{+0.39}_{-0.35}$ from Ref.[31] |
| 060729 | 0.272 | 0.433±0.18 | SMC | (U-band)[21] |
| 061121 | 0.226 | 0.92±0.30 | MW | (U-band)[21] |
| 080310 | 0.196 | 0.433±0.18 | SMC | (U-band)[21] |
| 080607 | 0.111 | 2.98±0.37 | MW | (Rc-band)[21] |
| 091029 | 0.08 | ... | ... | (U-band) no host galaxy extinction[23] |
| 100418A | 0.362 | 1.2±0.43 | SMC | (white-band)[21] |
| 110213A | 1.612 | ... | ... | (U-band) no host galaxy extinction[24] |
| 130831A | 0.223 | 0.103±0.0513 | MW | (U-band) low host galaxy extinction[25] |
| 171205A | 0.251 | 0.103 | MW | (U-band)[26] |

**Supplementary Table 5. Galactic and host galaxy extinctions of the 13 GRBs in our sample.** Column 1: GRB name, Columns 2 and 3: Galactic and host galaxy extinctions in the U band. The errors correspond to a significance of one sigma. Column 4: The extinction law used to compute the extragalactic reddening. Column 5: band passes used in our analyses and corresponding references.

| GRB name | S (15-150 keV) ($10^{-7}$ erg cm$^{-2}$) | $\alpha_\gamma$ | $T_{90}$ ($s$) |
|---|---|---|---|
| Class I | | | |
| 080607 | 240±0.0 | 1.31±0.04 | 79.0 |
| 091029 | 24±1.0 | 1.46±0.27 | 39.2 |
| 110213A | 59±4.0 | 1.83±0.12 | 48.0 |
| 130831A | 65±0.2 | 1.93±0.05 | 32.5 |
| Class II | | | |
| 060605 | 7.0±0.9 | 1.55±0.20 | 79.1 |
| 060614 | 204±3.6 | 2.02±0.04 | 108.7 |
| 060729 | 26±2.1 | 1.75±0.14 | 115.3 |
| 080310 | 23±2.0 | 2.32±0.16 | 365.0 |
| 100418A | 3.4±0.5 | 2.16±0.25 | 7.0 |
| 171205A | 36±3.0 | 1.41±0.14 | 189.4 |
| Class III | | | |
| 050319 | 13.1±1.5 | 2.02±0.19 | 152.5 |
| 060714 | 28.3±1.7 | 1.93±0.11 | 115.0 |
| 061121 | 137±2.0 | 1.41±0.03 | 81.3 |

**Supplementary Table 6. Prompt parameters of the 13 GRBs in our sample.** Columns 1 — 4 are the GRB names (ordered by classes I, II, III listed in Supplementary Tables 1, 2, 3 respectively), fluence, photon spectral index, and burst duration. The errors correspond to a significance of one sigma. Note that the fluence of GRB 080607 has no error in the online *Swift* GRB table. See, Supplementary Method 1c, Flux Ratio for the definition of each parameters.

| Scenario | Coasting | Self-Similar Decay |
|---|---|---|
| A $\nu < \nu_c < \nu_m$ | $F_\nu \propto t_{\rm obs.}^{-1/3} \nu^{1/3}$ | $F_\nu \propto t_{\rm obs.}^{-2/3} \nu^{1/3}$ |
| B $\nu_c < \nu < \nu_m$ | $F_\nu \propto t_{\rm obs.}^{1/2} \nu^{-1/2}$ | $F_\nu \propto t_{\rm obs.}^{-1/4} \nu^{-1/2}$ |
| C $\nu_c < \nu_m < \nu$ | $F_\nu \propto t_{\rm obs.}^{(2-p)/2} \nu^{-p/2} \sim t_{\rm obs.}^{0..-0.2}$ | $F_\nu \propto t_{\rm obs.}^{(2-3p)/4} \nu^{-p/2} \sim t_{\rm obs.}^{-1..-1.3}$ |
| D $\nu < \nu_m < \nu_c$ | $F_\nu \propto t_{\rm obs.}^{1/3} \nu^{1/3}$ | $F_\nu \propto t_{\rm obs.}^{0} \nu^{1/3}$ |
| E $\nu_m < \nu < \nu_c$ | $F_\nu \propto t_{\rm obs.}^{(1-p)/2} \nu^{-(p-1)/2} \sim t_{\rm obs.}^{-0.5..-0.7}$ | $F_\nu \propto t_{\rm obs.}^{(1-3p)/4} \nu^{-(p-1)/2} \sim t_{\rm obs.}^{-1.25..-1.55}$ |
| F $\nu_m < \nu_c < \nu$ | $F_\nu \propto t_{\rm obs.}^{(2-p)/2} \nu^{-p/2} \sim t_{\rm obs.}^{0..-0.2}$ | $F_\nu \propto t_{\rm obs.}^{(2-3p)/4} \nu^{-p/2} \sim t_{\rm obs.}^{-1..-1.3}$ |

**Supplementary Table 7. Temporal and spectral evolution in each of the possible six spectral regimes with the corresponding regions indicated in the first column**. Here, $p$ is the power-law index of the accelerated electrons. Expected values based on both theory[37] and observations[38] suggest $2.0 \leq p \lesssim 2.4$. We indicate the expected temporal evolution in regions C, E and F for power-law index in this range. For the definition of each parameters, see Supplementary Method 2, Theoretical model.

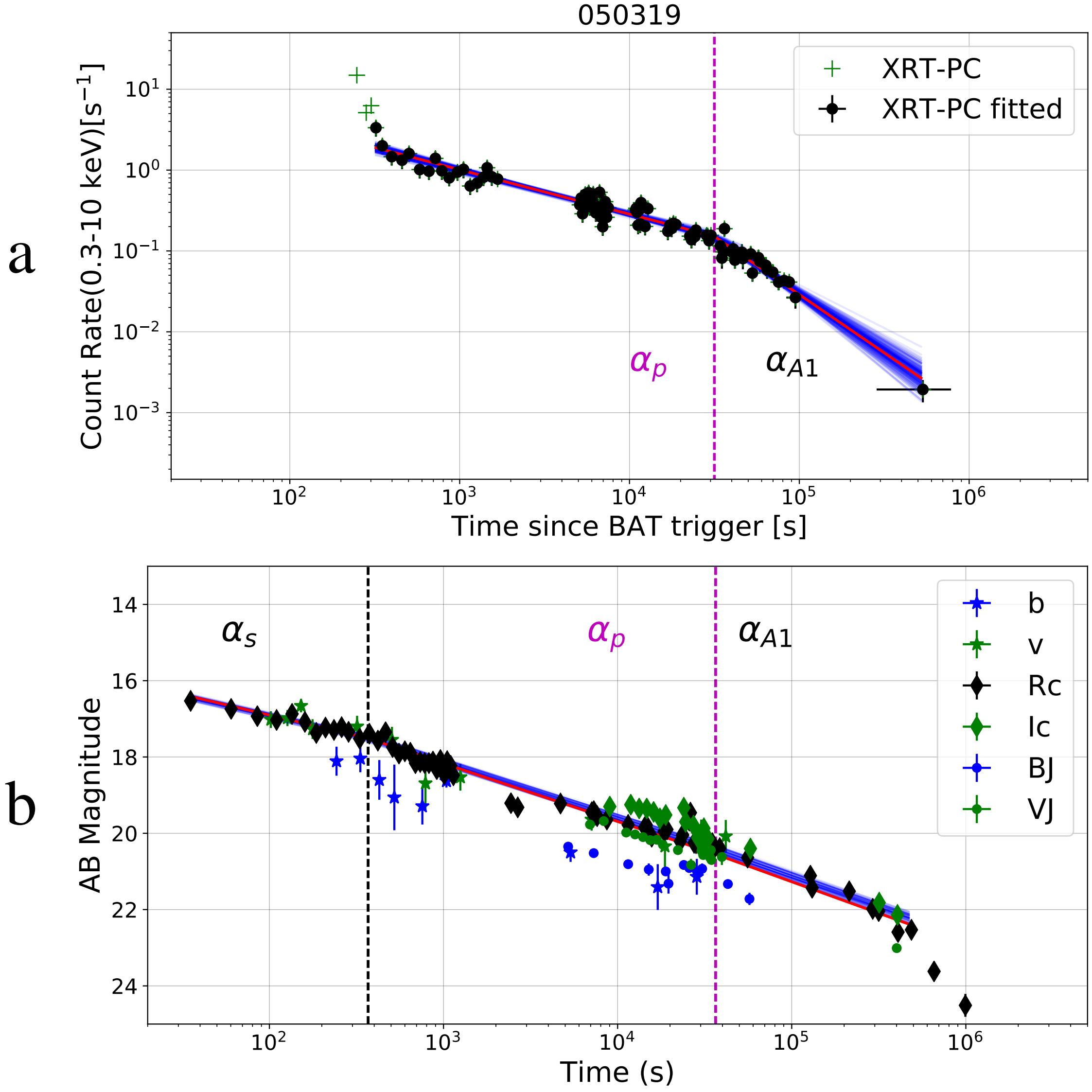


**Supplementary Figure 1.** **(a) The X-ray LC of GRB 050319.** The green crosses represent the XRT-PC mode data which are excluded from the fit. The black dots represent XRT-PC mode fitted data. The errors correspond to a significance of one sigma. The dashed vertical (purple) line represents the break times $T_{\mathrm{a}}$ (at the end of the plateau phase). The $\alpha_{\mathrm{p}}$ and $\alpha_{\mathrm{A1}}$ are the slopes during the plateau phase and the self-similar phase respectively. **(b) The optical LC of GRB 050319.** The blue and green colors together with stars, diamonds and dots represent the data in b, v, Rc, Ic, BJ, VJ bands respectively. The errors correspond to a significance of one sigma. For the details of the excluded data, see, Supplementary Method 1b, Optical data and fitting process. Although all the bands are jointly fitted, for the demonstration purpose, only the Rc band fit result is displayed. The dashed vertical lines (black and purple) represent the break times $T_{\mathrm{s}}$ and $T_{\mathrm{a}}$ (at the end of the plateau phase) respectively. The $\alpha_{\mathrm{As}}$, $\alpha_{\mathrm{p}}$, $\alpha_{\mathrm{A1}}$, are the slopes before the plateau phase, during the plateau phase and self-similar phase respectively. In both panels **(a)** and **(b)**, the red line shows the mean of the posterior distribution and blue lines are 200 randomly selected samples from the MCMC sampling.

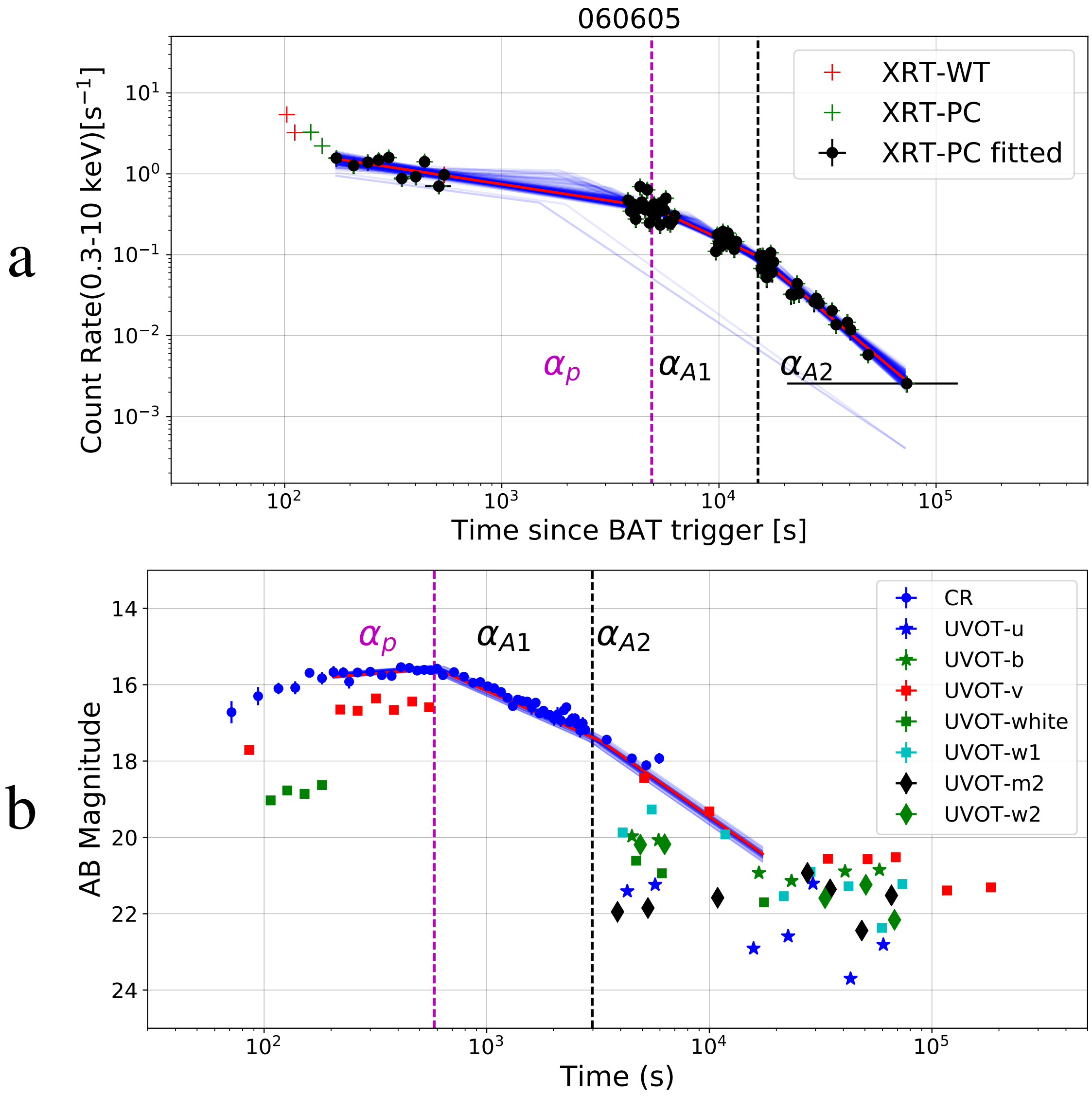


**Supplementary Figure 2. (a) The X-ray LC of GRB 060605.** The red and green crosses represent the XRT-WT and the XRT-PC mode data respectively which are excluded from the fit. The black dots represent the XRT-PC mode fitted data. The errors correspond to a significance of one sigma. **(b) The optical LC of GRB 060605.** The blue, green, red, cyan, black colors together with points, stars, squares diamonds represent the data in CR, UVOT-u, UVOT-b, UVOT-v, UVOT-white, UVOT-w1, UVOT-m2, UVOT-w2 bands respectively. The errors correspond to a significance of one sigma. For the details of the excluded data, see, Supplementary Method 1b, Optical data and fitting process. Although all the bands jointly fitted, for the demonstration purpose only CR band fit result displayed. In both panels **(a)** and **(b)**, the red line shows the mean of the posterior distribution and blue lines are 200 randomly selected samples from the MCMC sampling. The dashed vertical lines (purple and black) represent the break times $T_a$ (at the end of the plateau phase) and $T_b$ respectively. The $\alpha_p$, $\alpha_{A1}$, $\alpha_{A2}$ are slopes during the plateau phase, self-similar phase and after the jet break time respectively.

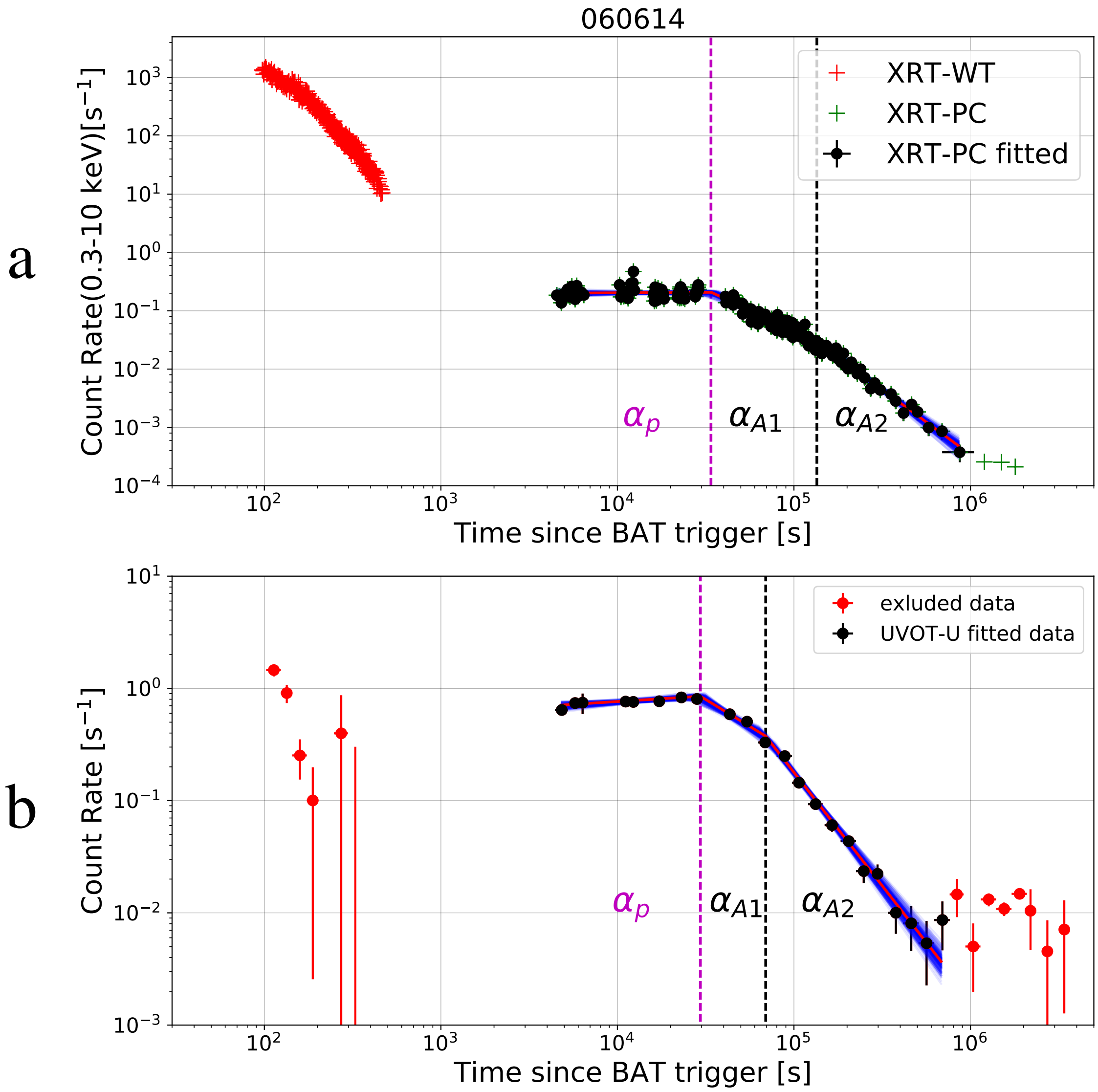


**Supplementary Figure 3.** **(a) The X-ray LC of GRB 060614.** The red and green crosses represent the XRT-WT and the XRT-PC mode data respectively which are excluded from the fit. The black dots represent the XRT-PC mode fitted data. The errors correspond to a significance of one sigma. **(b) The optical LC of GRB 060614.** The black and red dots represent the data in UVOT-u band. The errors correspond to a significance of one sigma. While the black dots are considered in the fitting process, the red dots are excluded from the fit (see, Supplementary Method 1b, Optical data and fitting process for explanations). In both panels **(a)** and **(b)**, the red line shows the mean of the posterior distribution and blue lines are 200 randomly selected samples from the MCMC sampling. The dashed vertical lines (purple and black) represent the break times $T_a$ (at the end of the plateau phase) and $T_b$ respectively. The $\alpha_p$, $\alpha_{A1}$, $\alpha_{A2}$ are slopes during the plateau phase, self-similar phase and after the jet break time respectively.

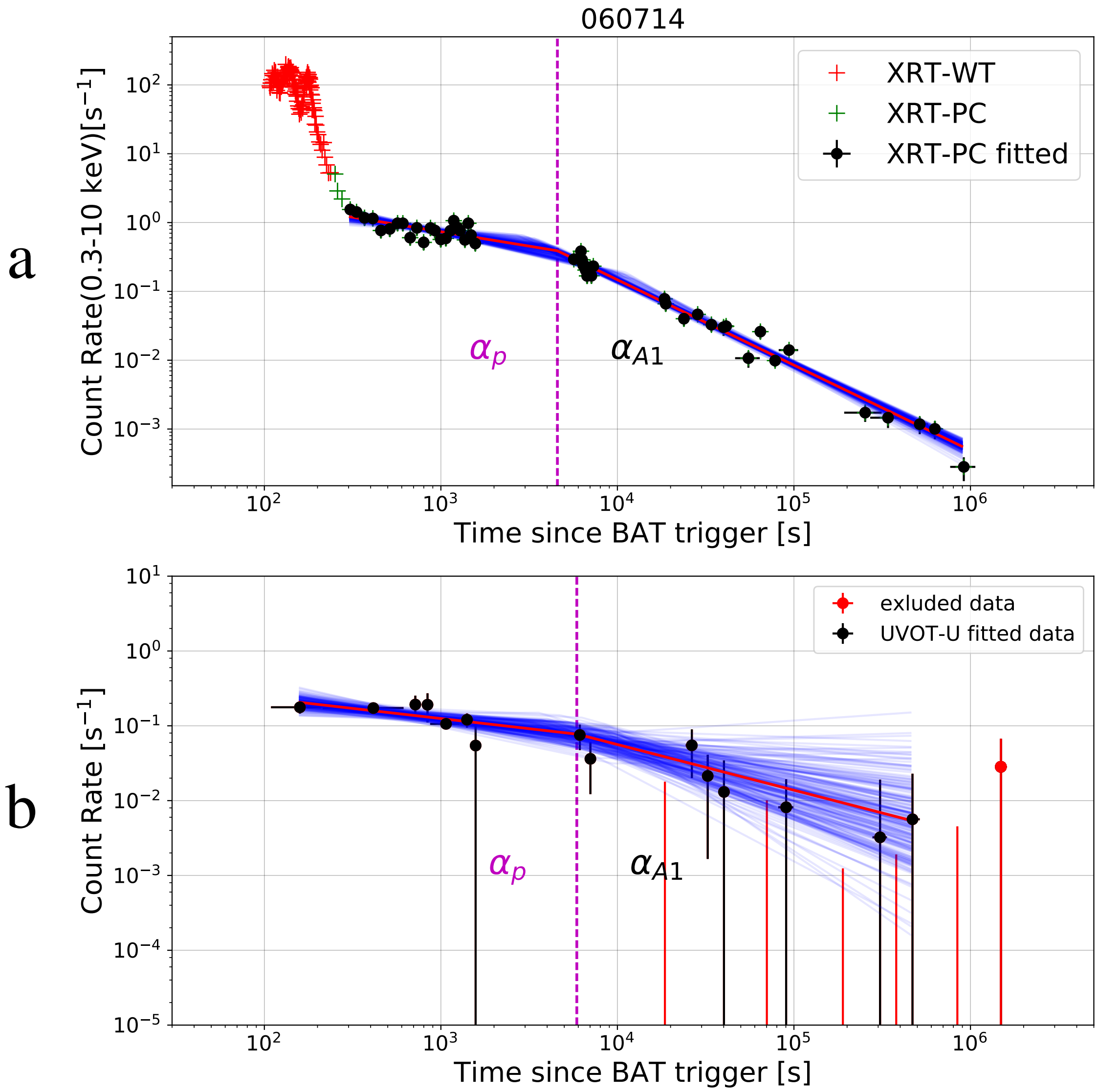


**Supplementary Figure 4. (a) The X-ray LC of GRB 060714.** The red and green crosses represent the XRT-WT and the XRT-PC mode data respectively which are excluded from the fit. The black dots represent the XRT-PC mode fitted data. The errors correspond to a significance of one sigma. **(b) The optical LC of GRB 060714.** The black and red dots represent the data in UVOT-u band. The errors correspond to a significance of one sigma. While the black dots are considered in the fitting process, the red dots are excluded from the fit (see, Supplementary Method 1b, Optical data and fitting process for explanations). In both panels **(a)** and **(b)**, the red line shows the mean of the posterior distribution and blue lines are 200 randomly selected samples from the MCMC sampling. The dashed vertical line (purple) represents the break times $T_{\mathrm{a}}$ (at the end of the plateau phase). The $\alpha_{\mathrm{p}}$, $\alpha_{\mathrm{A1}}$ are slopes during the plateau phase and self-similar phase respectively.

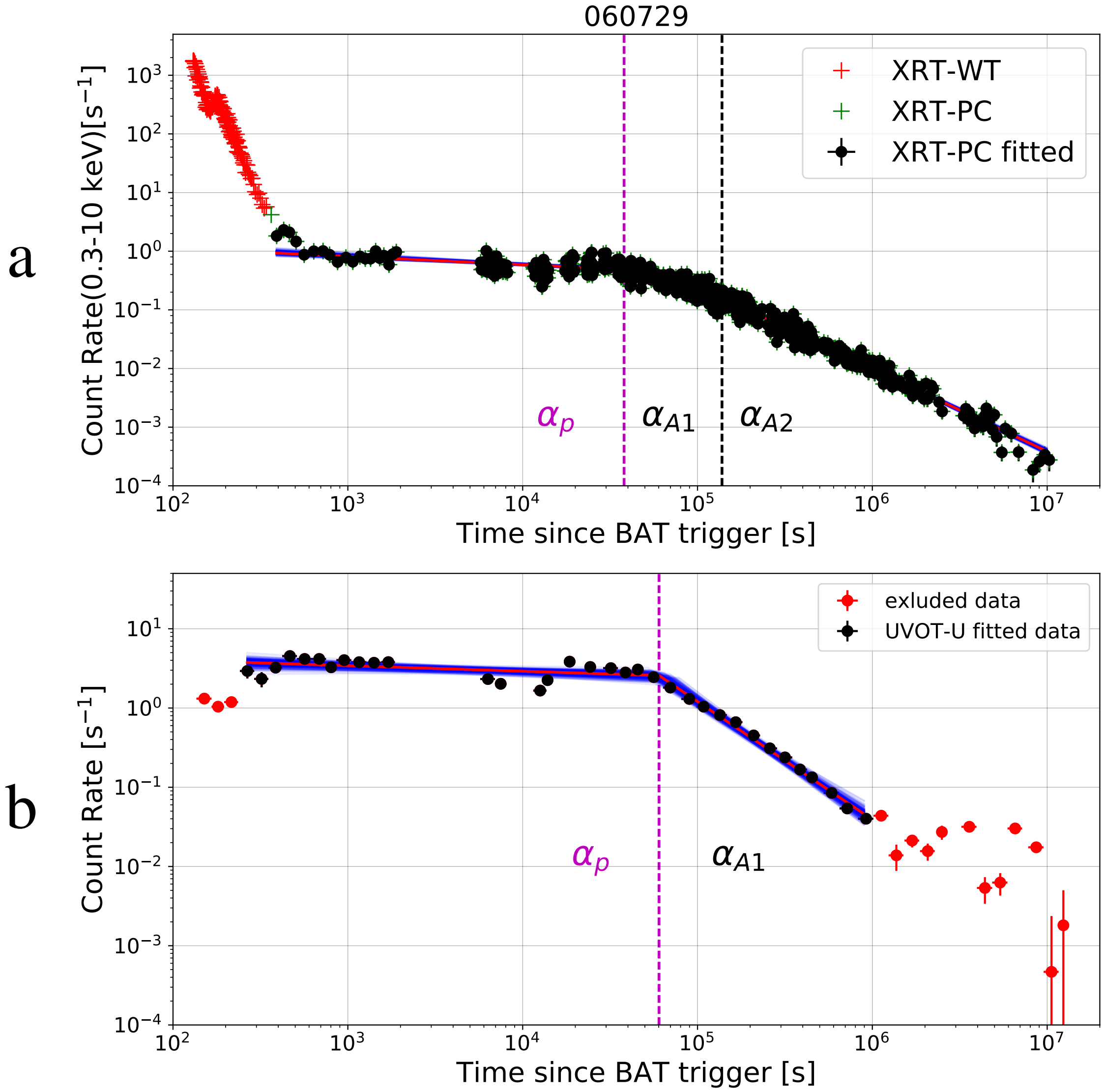


**Supplementary Figure 5. (a) The X-ray LC of GRB 060614.** The red and green crosses represent the XRT-WT and the XRT-PC mode data respectively which are excluded from the fit. The black dots represent the XRT-PC mode fitted data. The errors correspond to a significance of one sigma. The dashed vertical lines (purple and black) represent the break times $T_a$ (at the end of the plateau phase) and $T_b$ respectively. The $\alpha_p$, $\alpha_{A1}$, $\alpha_{A2}$ are slopes during the plateau phase, self-similar phase and after the jet break time respectively. **(b) The optical LC of GRB 060614.** The black and red dots represent the data in UVOT-u band. The errors correspond to a significance of one sigma. While the black dots are considered in the fitting process, the red dots are excluded from the fit (see, Supplementary Method 1b, Optical data and fitting process for explanations). The dashed vertical line (purple) represents the break times $T_a$ (at the end of the plateau phase). The $\alpha_p$, $\alpha_{A1}$ are slopes during the plateau phase and self-similar phase respectively. In both panels **(a)** and **(b)**, the red line shows the mean of the posterior distribution and blue lines are 200 randomly selected samples from the MCMC sampling.

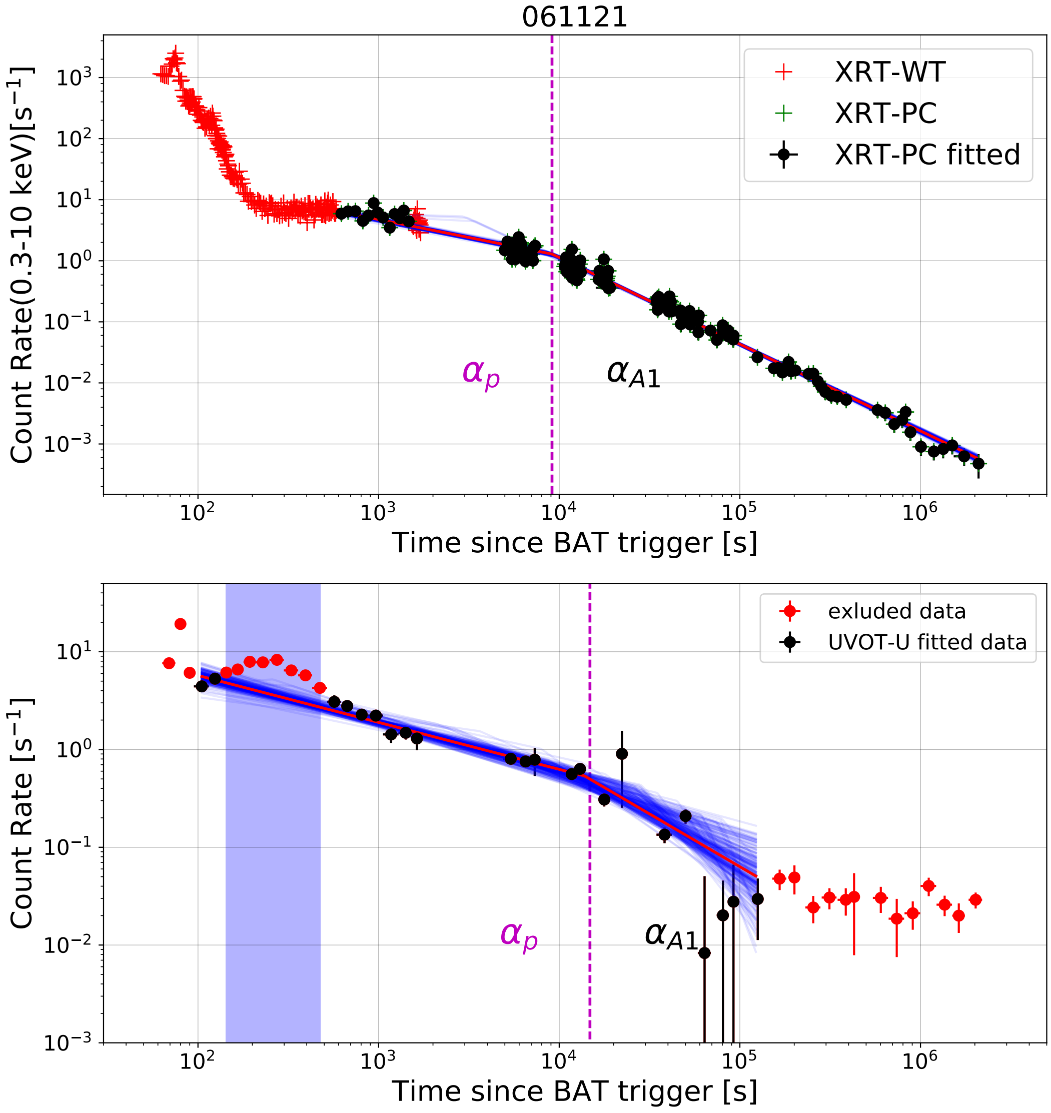


**Supplementary Figure 6.** **(a) The X-ray LC of GRB 061121.** The red crosses represent the XRT-WT mode data which are excluded from the fit. The black dots represent the XRT-PC mode fitted data. The errors correspond to a significance of one sigma. **(b) The optical LC of GRB 061121.** The black and red dots represent the data in UVOT-u band.The errors correspond to a significance of one sigma. While the black dots are considered in the fitting process, the red dots (including blue shaded region) are excluded from the fit (see, Supplementary Method 1b, Optical data and fitting process for explanations). In both panels **(a)** and **(b)**, the red line shows the mean of the posterior distribution and blue lines are 200 randomly selected samples from the MCMC sampling. The dashed vertical line (purple) represents the break times $T_a$ (at the end of the plateau phase). The $\alpha_p$, $\alpha_{A1}$ are slopes during the plateau phase and self-similar phase respectively.

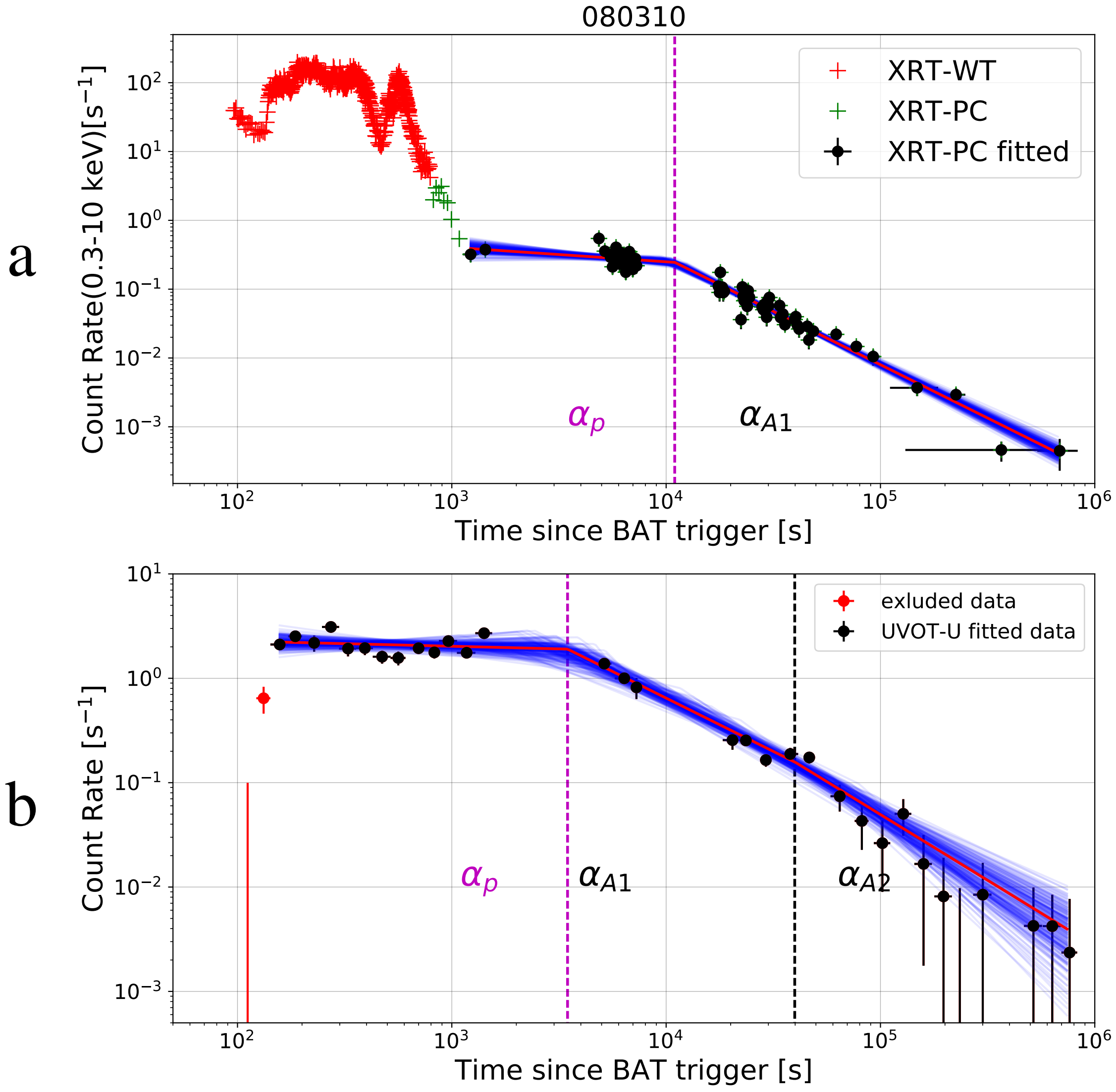


**Supplementary Figure 7.** **(a) The X-ray LC of GRB 061121.** The red crosses represent the XRT-WT and XRT-PC mode data respectively which are excluded from the fit. The black dots represent the XRT-PC mode fitted data. The errors correspond to a significance of one sigma. The dashed vertical line (purple) represents the break times $T_a$ (at the end of the plateau phase). The $\alpha_p$, $\alpha_{A1}$ are slopes during the plateau phase and self-similar phase respectively. **(b) The optical LC of GRB 061121.** The black and red dots represent the data in UVOT-u band. The errors correspond to a significance of one sigma. While the black dots are considered in the fitting process, the red dots (including blue shaded region) are excluded from the fit (see, Supplementary Method 1b, Optical data and fitting process for explanations). The dashed vertical lines (purple and black) represent the break times $T_a$ (at the end of the plateau phase) and $T_b$ respectively. The $\alpha_p$, $\alpha_{A1}$, $\alpha_{A2}$ are slopes during the plateau phase, self-similar phase and after the jet break time respectively. In both panels **(a)** and **(b)**, the red line shows the mean of the posterior distribution and blue lines are 200 randomly selected samples from the MCMC sampling.

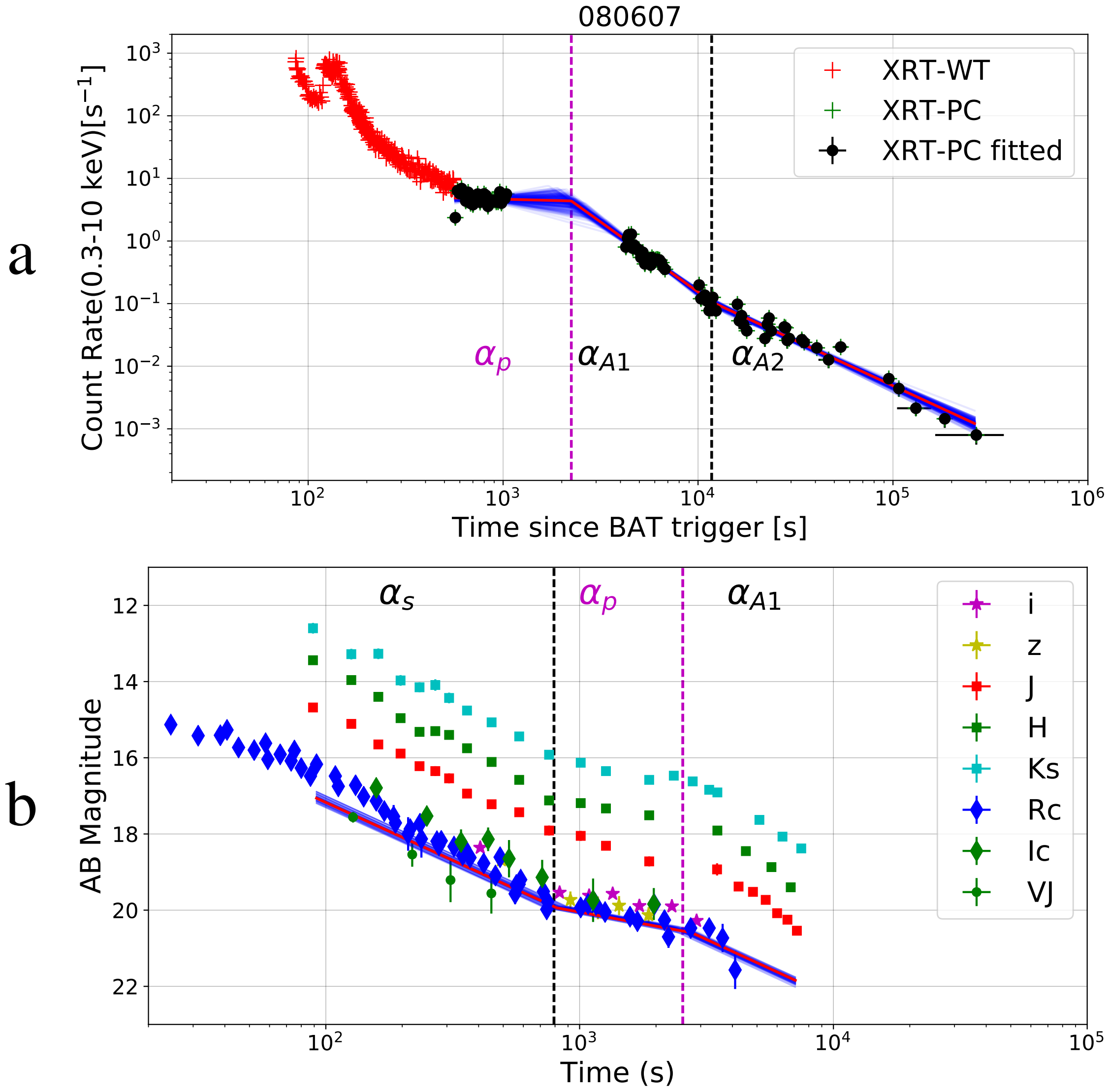


**Supplementary Figure 8.** **(a) The X-ray LC of GRB 080607.** The red crosses represent the XRT-WT mode data which are excluded from the fit. The black dots represent XRT-PC mode fitted data. The errors correspond to a significance of one sigma. The dashed vertical lines (purple and black) represent the break times $T_{\mathrm{a}}$ (at the end of the plateau phase) and $T_{\mathrm{b}}$ respectively. The $\alpha_{\mathrm{p}}$, $\alpha_{\mathrm{A1}}$, $\alpha_{\mathrm{A2}}$ are slopes during the plateau phase, self-similar phase and after the jet break time respectively. **(b) The optical LC of GRB 080607.** The purple, yellow, red, green, cyan, blue, black colors together with stars, squares, diamonds and point represent the data in i, z, J, H, K, Rc, Ic, VJ bands respectively. The errors correspond to a significance of one sigma. For the details of the excluded data, see, Supplementary Method 1b, Optical data and fitting process. Although all the bands are jointly fitted, for the demonstration purpose, only the Rc band fit result is displayed. The dashed vertical lines (black and purple) represent the break times $T_{\mathrm{s}}$ and $T_{\mathrm{a}}$ (at the end of the plateau phase) respectively. The $\alpha_{\mathrm{As}}$, $\alpha_{\mathrm{p}}$, $\alpha_{\mathrm{A1}}$, are the slopes before the plateau phase, during the plateau phase and self-similar phase respectively. In both panels **(a)** and **(b)**, the red line shows the mean of the posterior distribution and blue lines are 200 randomly selected samples from the MCMC sampling.

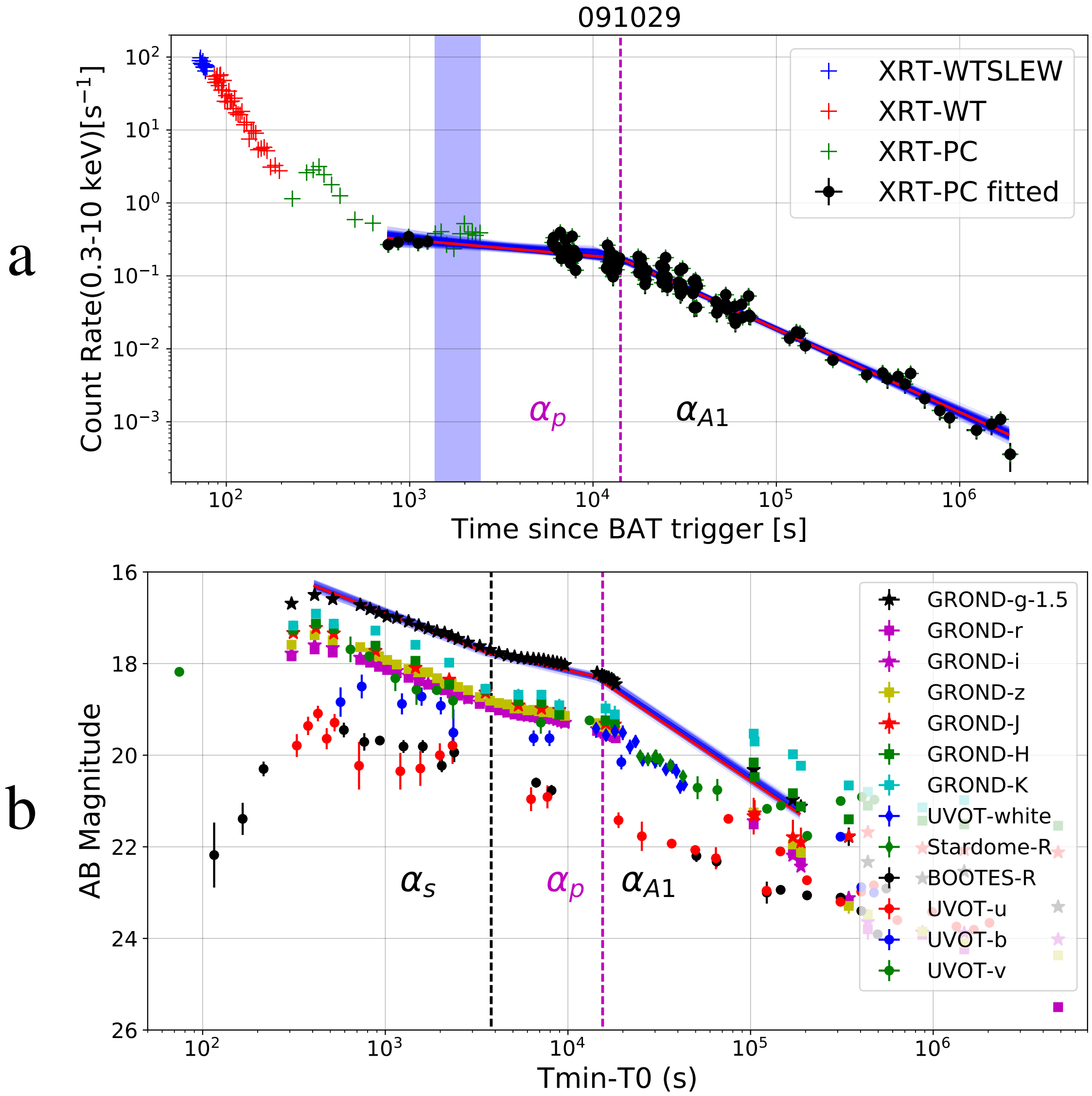


**Supplementary Figure 9.** **(a) The X-ray LC of GRB 091029.** The blue, red and green crosses represent the XRT-WTSLEW, XRT-WT, XRT-PC (including blue shaded region, see, Supplementary Method 1a, X-ray data and fitting process for explanation) mode data which are excluded from the fit. The black dots represent XRT-PC mode fitted data. The errors correspond to a significance of one sigma. The dashed vertical line (purple) represents the break times $T_a$ (at the end of the plateau phase). The $\alpha_p$, $\alpha_{A1}$ are slopes during the plateau phase and self-similar phase respectively. **(b) The optical LC of GRB 091029.** The black, purple, yellow, red, green, cyan, blue, black colors together with stars, squares, diamonds and points represent the data in GROND-g, GROND-r, GROND-i, GROND-z, GROND-J, GROND-H, GROND-K, UVOT-white, Stardome-R, BOOTES-R, UVOT-u, UVOT-b, UVOT-v bands respectively. The errors correspond to a significance of one sigma. For the details of the excluded data, see, Supplementary Method 1b, Optical data and fitting process. Although all the bands are jointly fitted, for the demonstration purpose, only the GROND-g band fit result is displayed and GROND-g band light curve distinguished by subtracting 1.5 from the AB magnitude. The dashed vertical lines (black and purple) represent the break times $T_s$ and $T_a$ (at the end of the plateau phase) respectively. The $\alpha_{As}$, $\alpha_p$, $\alpha_{A1}$, are the slopes before the plateau phase, during the plateau phase and self-similar phase respectively. In both panels **(a)** and **(b)**, the red line shows the mean of the posterior distribution and blue lines are 200 randomly selected samples from the MCMC sampling.

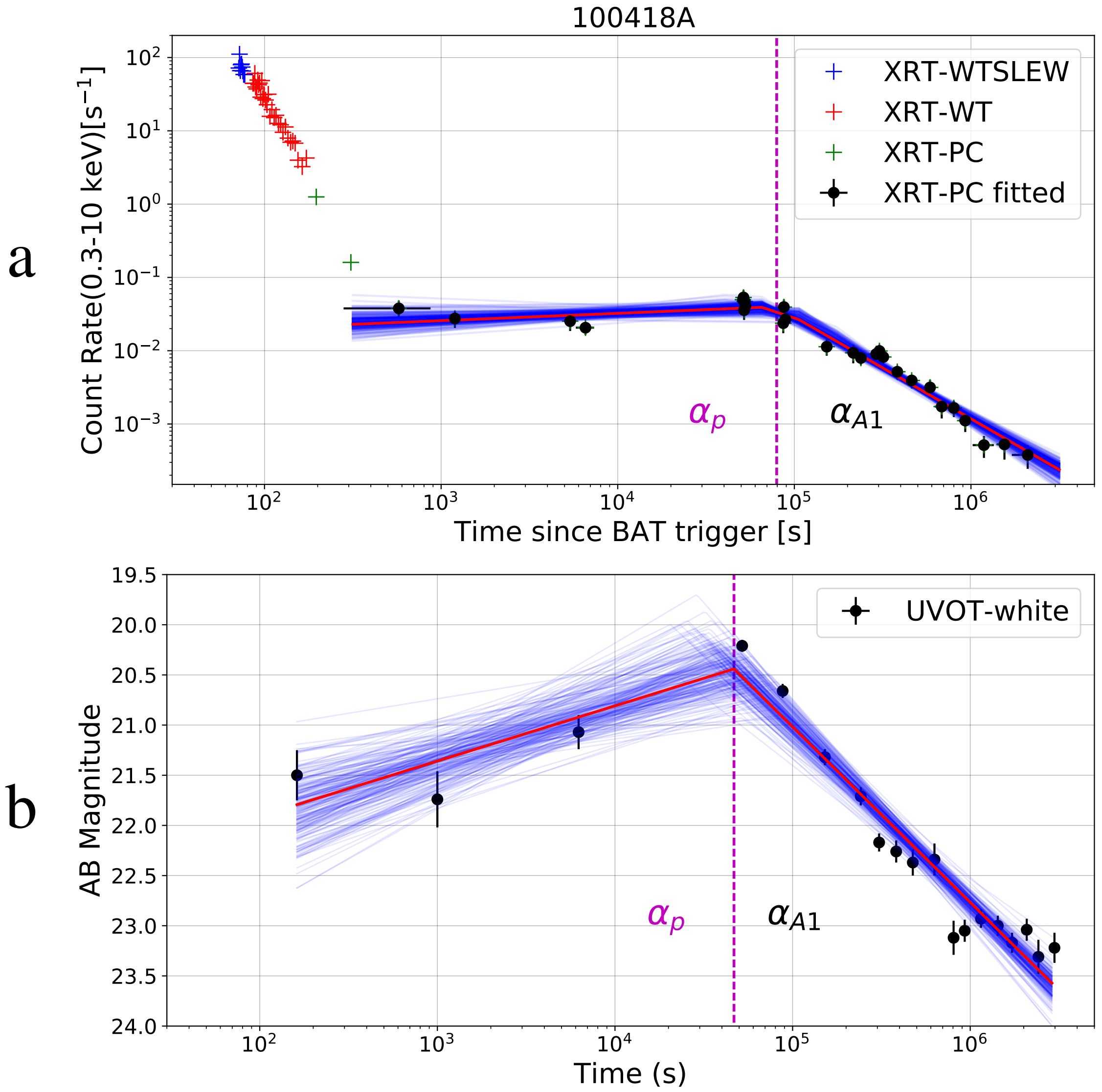


**Supplementary Figure 10.** **(a) The X-ray LC of GRB 100418A.** The blue, red and green crosses represent the XRT-WTSLEW, XRT-WT, XRT-PC mode data which are excluded from the fit. The black dots represent XRT-PC mode fitted data. The errors correspond to a significance of one sigma. **(b) The optical LC of GRB 100418A.** The black points represent the data in UVOT-white band. The errors correspond to a significance of one sigma. In both panels **(a)** and **(b)**, the red line shows the mean of the posterior distribution and blue lines are 200 randomly selected samples from the MCMC sampling. The dashed vertical line (purple) represents the break times $T_a$ (at the end of the plateau phase). The $\alpha_p$, $\alpha_{A1}$ are slopes during the plateau phase and self-similar phase respectively.

a

b

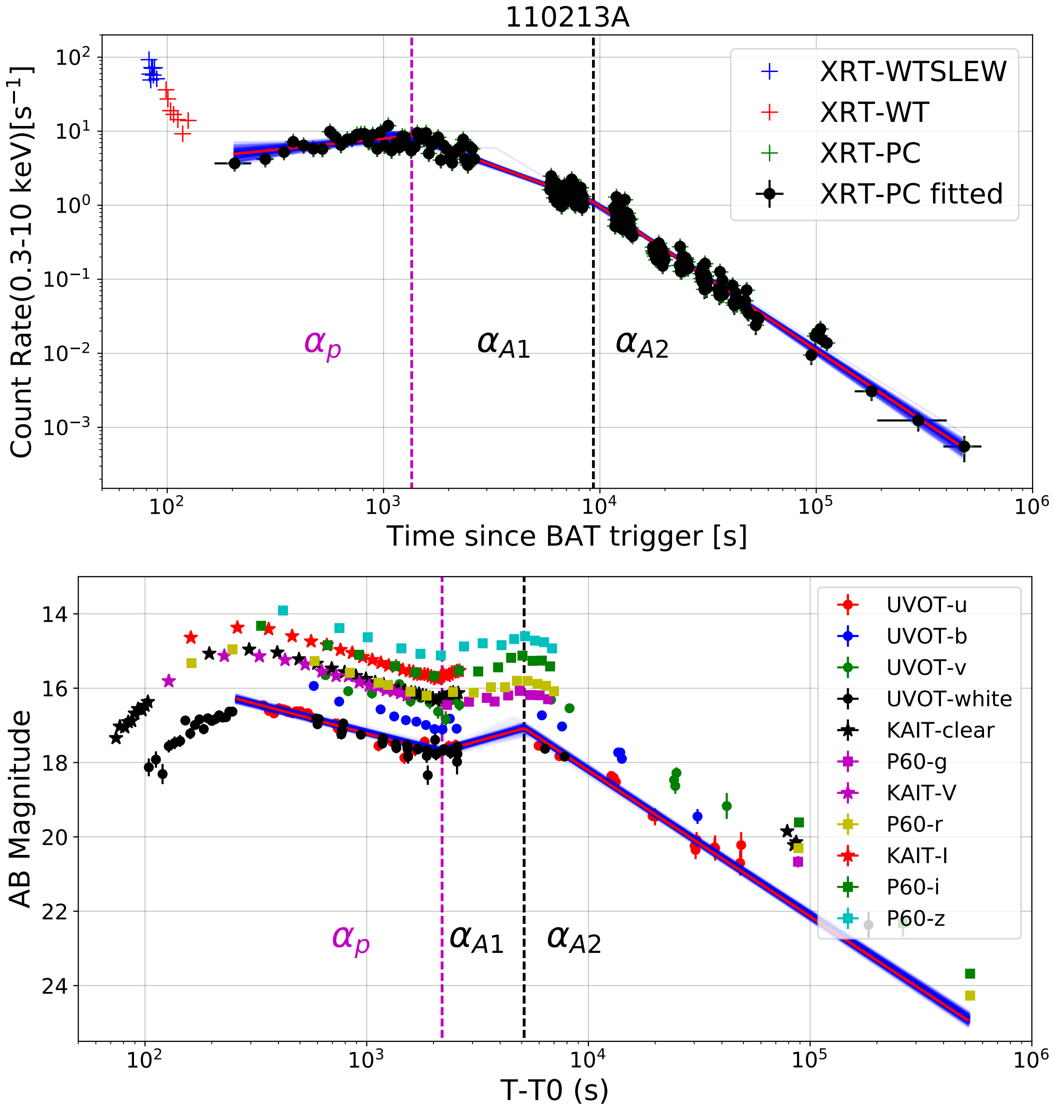


**Supplementary Figure 11.** **(a) The X-ray LC of GRB 110213A.** The blue, red and green crosses represent the XRT-WTSLEW and XRT-WT mode data respectively which are excluded from the fit. The black dots represent the XRT-PC mode fitted data. The errors correspond to a significance of one sigma. **(b) The optical LC of GRB 110213A.** The red, blue, green, black, purple, yellow, cyan colors together with points, stars, squares represent the data in UVOT-u, UVOT-b, UVOT-v, UVOT-white, KAIT-clear, P60-g, KAIT-V, P60-r, KAIT-I, P60-i, P60-z bands respectively. The errors correspond to a significance of one sigma. For the details of the excluded data, see, Supplementary Method 1b, Optical data and fitting process. Although all the bands jointly fitted, for the demonstration purpose only UVOT-u band fit result displayed. In both panels **(a)** and **(b)**, The red line shows the mean of the posterior distribution and blue lines are 200 randomly selected samples from the MCMC sampling. The dashed vertical lines (purple and black) represent the break times $T_a$ (at the end of the plateau phase) and $T_b$ respectively. The $\alpha_p$, $\alpha_{A1}$, $\alpha_{A2}$ are slopes during the plateau phase, self-similar phase and after the jet break time respectively.

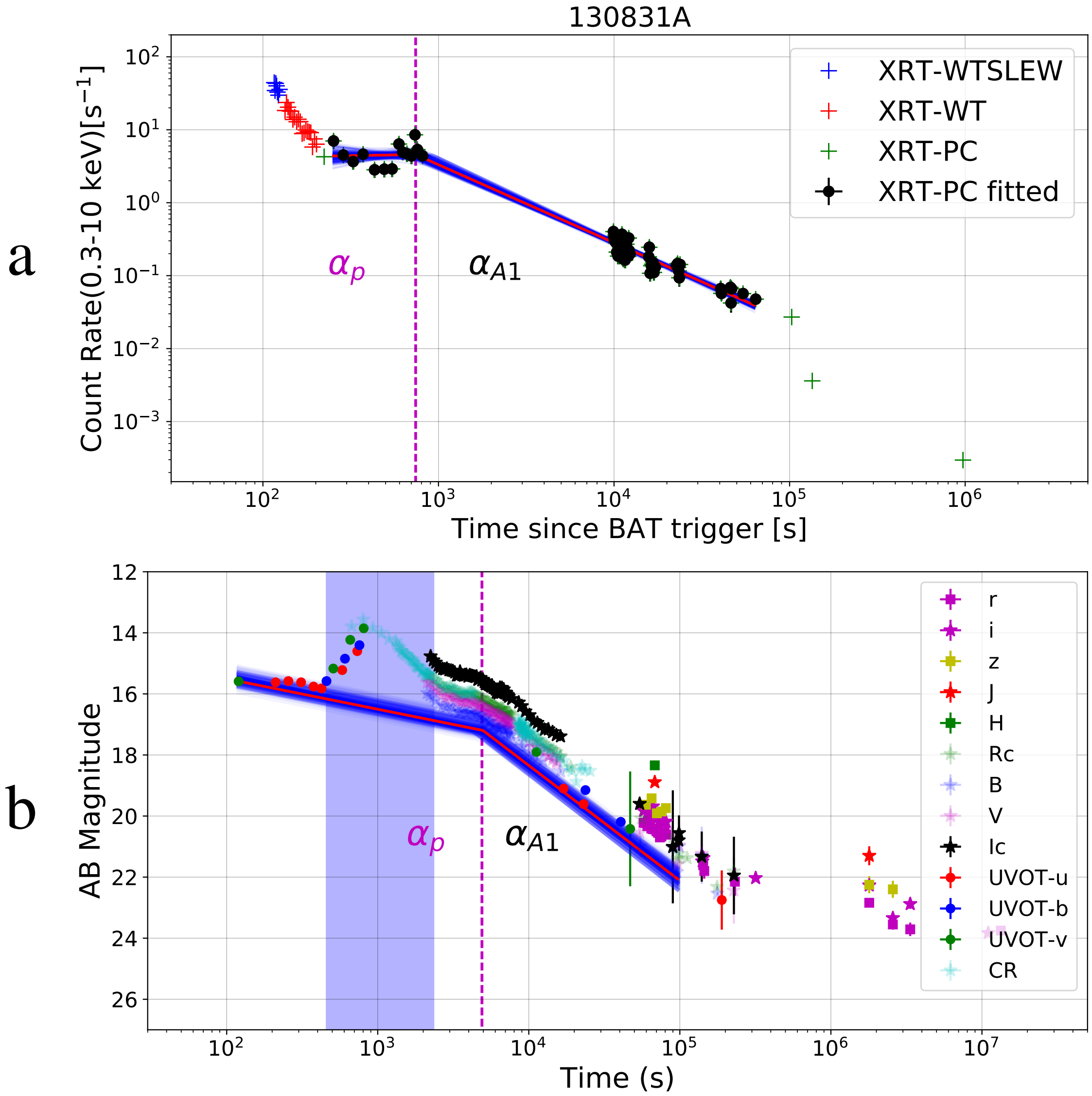


**Supplementary Figure 12.** **(a) The X-ray LC of GRB 130831A.** The blue, red and green crosses represent the XRT-WTSLEW, XRT-WT, XRT-PC mode data which are excluded from the fit. The black dots represent XRT-PC mode fitted data. The errors correspond to a significance of one sigma. **(b) The optical LC of GRB 130831A.** The purple, yellow, red, green, blue, black, cyan colors together with squares, stars and points represent the data in r, i, z, J, H, Rc, B, V, Ic, UVOT-u, UVOT-b, UVOT-v and CR bands respectively. The errors correspond to a significance of one sigma. The data marked by blue shaded region excluded from the fit, see, Supplementary Method 1b, Optical data and fitting process for explanation. Although all the bands jointly fitted, for the demonstration purpose only UVOT-u band fit result displayed. In both panels **(a)** and **(b)**, the red line shows the mean of the posterior distribution and blue lines are 200 randomly selected samples from the MCMC sampling. The dashed vertical line (purple) represents the break times $T_a$ (at the end of the plateau phase). The $\alpha_p$, $\alpha_{A1}$ are slopes during the plateau phase and self-similar phase respectively.

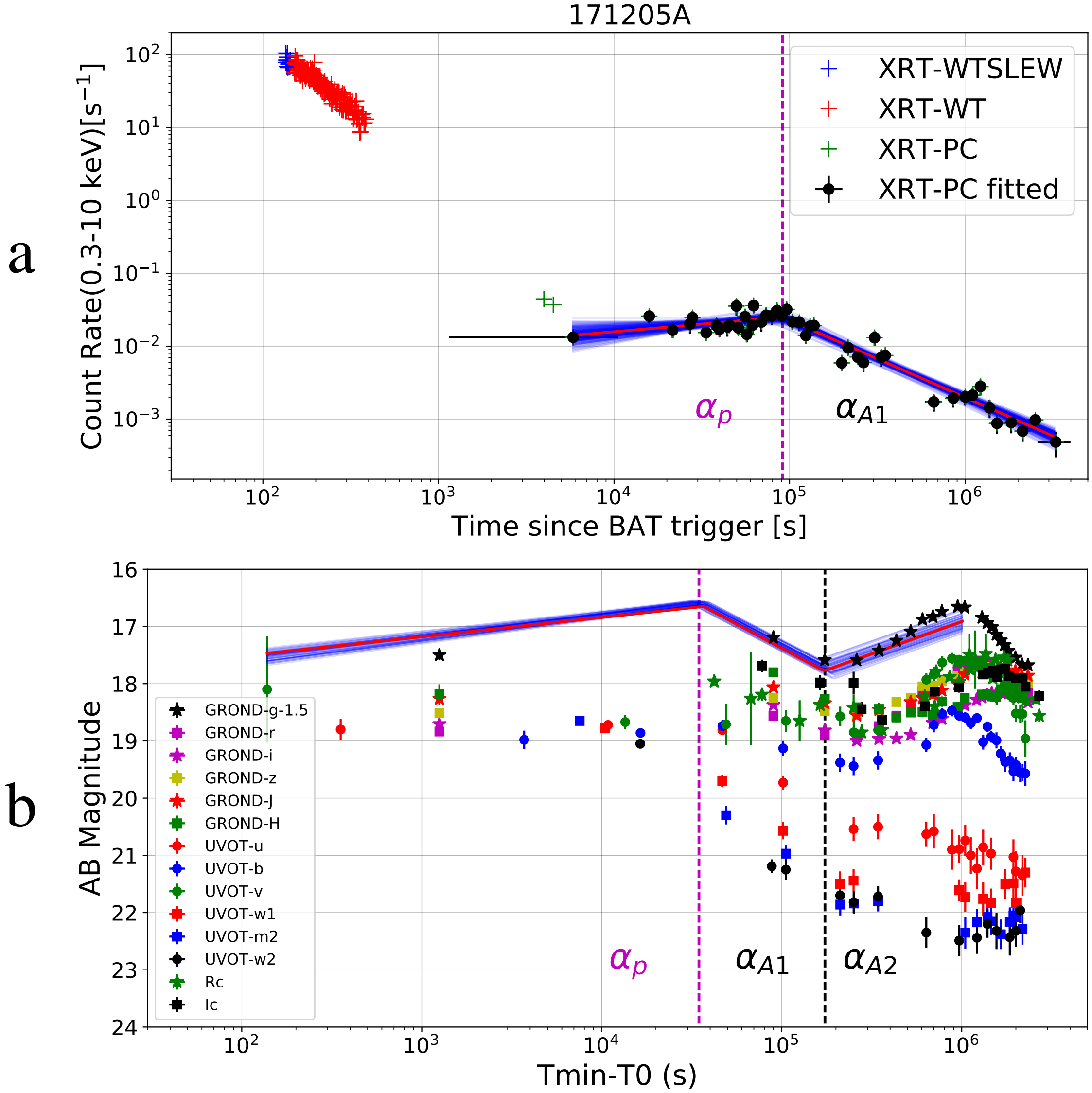


**Supplementary Figure 13.** **(a) The X-ray LC of GRB 171205A.** The blue, red and green crosses represent the XRT-WTSLEW, XRT-WT, XRT-PC mode data which are excluded from the fit. The black dots represent XRT-PC mode fitted data. The errors correspond to a significance of one sigma. The dashed vertical line (purple) represents the break times $T_a$ (at the end of the plateau phase). The $\alpha_p$, $\alpha_{A1}$ are slopes during the plateau phase and self-similar phase respectively. **(b) The optical LC of GRB 171205A.** The black, purple, yellow, red, green, blue colors together with points, stars, squares represent the data GROND-g, GROND-r, GROND-i, GROND-z, GROND-J, GROND-H, UVOT-u, UVOT-b, UVOT-v, UVOT-w1, UVOT-m2, UVOT-w2, Rc, Ic bands respectively. The errors correspond to a significance of one sigma. The cyan colors represent the other data that are not considered in the fitting process. For the details of the excluded data, see, Supplementary Method 1b, Optical data and fitting process. Although all the bands are jointly fitted, for the demonstration purpose, only the GROND-g band fit result is displayed and GROND-g band light curve distinguished by subtracting 1.5 from the AB magnitude. The dashed vertical lines (black and purple) represent the break times $T_s$ and $T_a$ (at the end of the plateau phase) respectively. The $\alpha_{As}$, $\alpha_p$, $\alpha_{A1}$, are the slopes before the plateau phase, during the plateau phase and self-similar phase respectively. In both panels **(a)** and **(b)**, the red line shows the mean of the posterior distribution and blue lines are 200 randomly selected samples from the MCMC sampling.

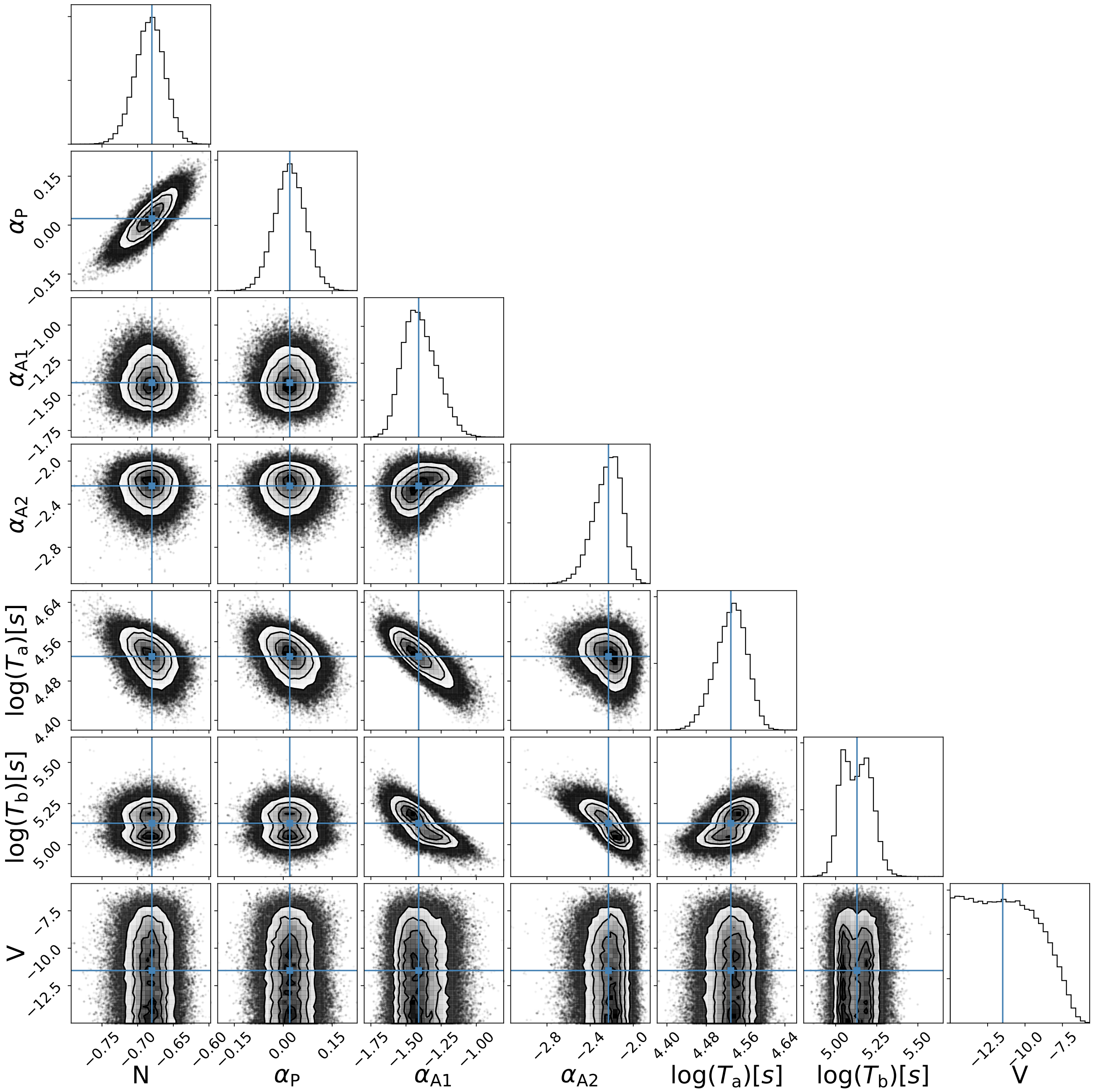


**Supplementary Figure 14. Corner plot showing the one-and two-dimensional posterior probability distributions for the fit parameters obtained from the X-ray LC of GRB 060614.** The blue lines present the mean value of each parameter given in Supplementary Table 4. Contours gives from inward to outward 39.3%, 68.3% ,90.3% confidence region.

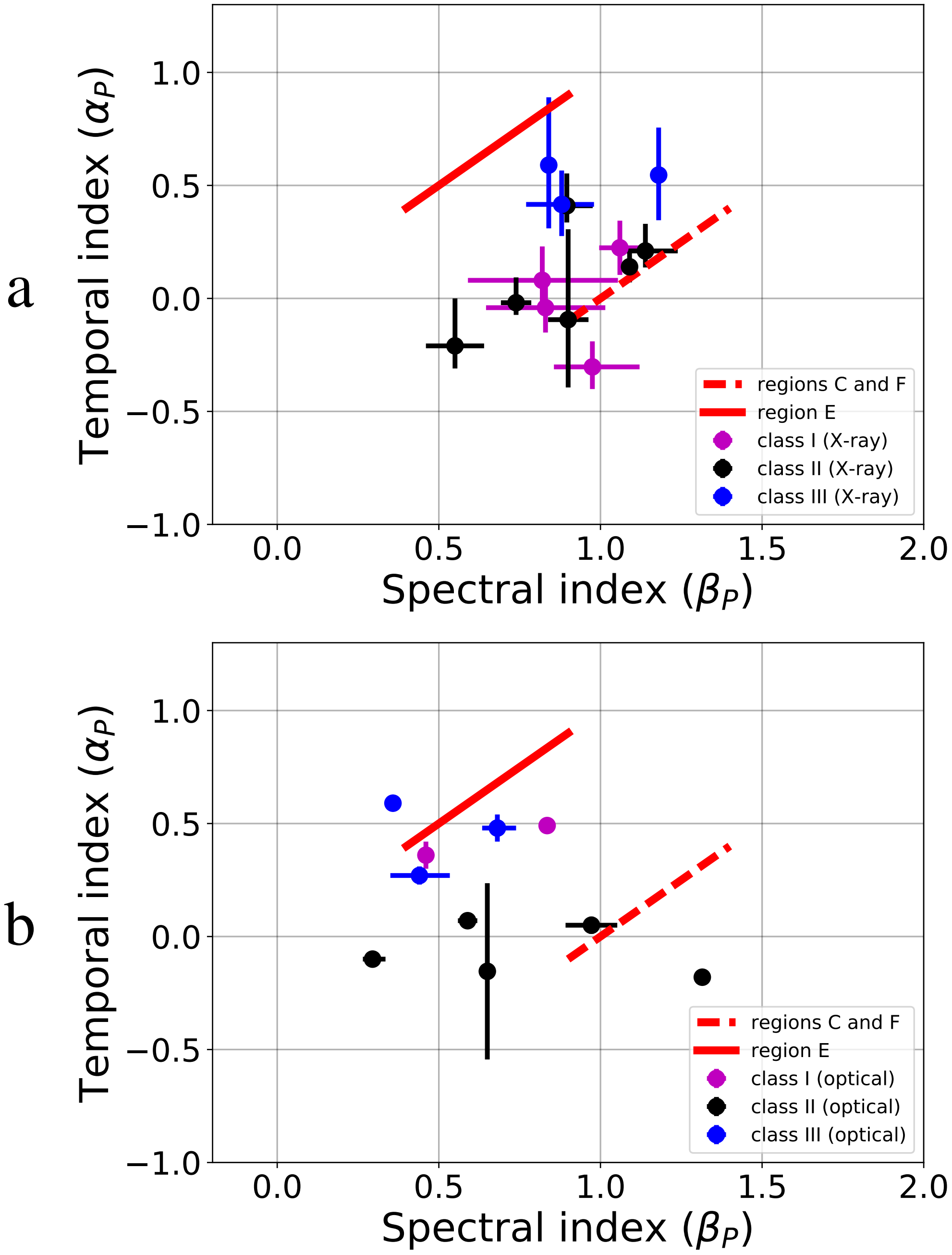


**Supplementary Figure 15. Temporal and spectral indices of the 13 GRBs in our sample during the plateau phase (a) in the X-ray band and (b) in the optical band.** The purple, black, and blue data points correspond to the three classes (I, II and III respectively). The errors correspond to a significance of one sigma. The spectral indices in the optical band of GRBs 100418A (in class II), 110213A and 130831A (both in class I) during the plateau phase could not be retrieved from the literature, and therefore these GRBs are excluded from panel **(b)**. The red line shows the closure relation obtained for region E in which $\nu_m < \nu_{\rm obs.} < \nu_c$, and the dashed red line represents regions C and F in which $\nu_c < \nu_m < \nu_{\rm obs.}$ and $\nu_m < \nu_c < \nu_{\rm obs.}$ respectively. These lines are computed by using an electron power-law index $p = 1.8 - 2.8$. Despite the simplicity of the model, most data points are in between the expected theoretical limits in both X-ray and optic bands. The source data necessary to reproduce this Figure are provided as a Source Data file.

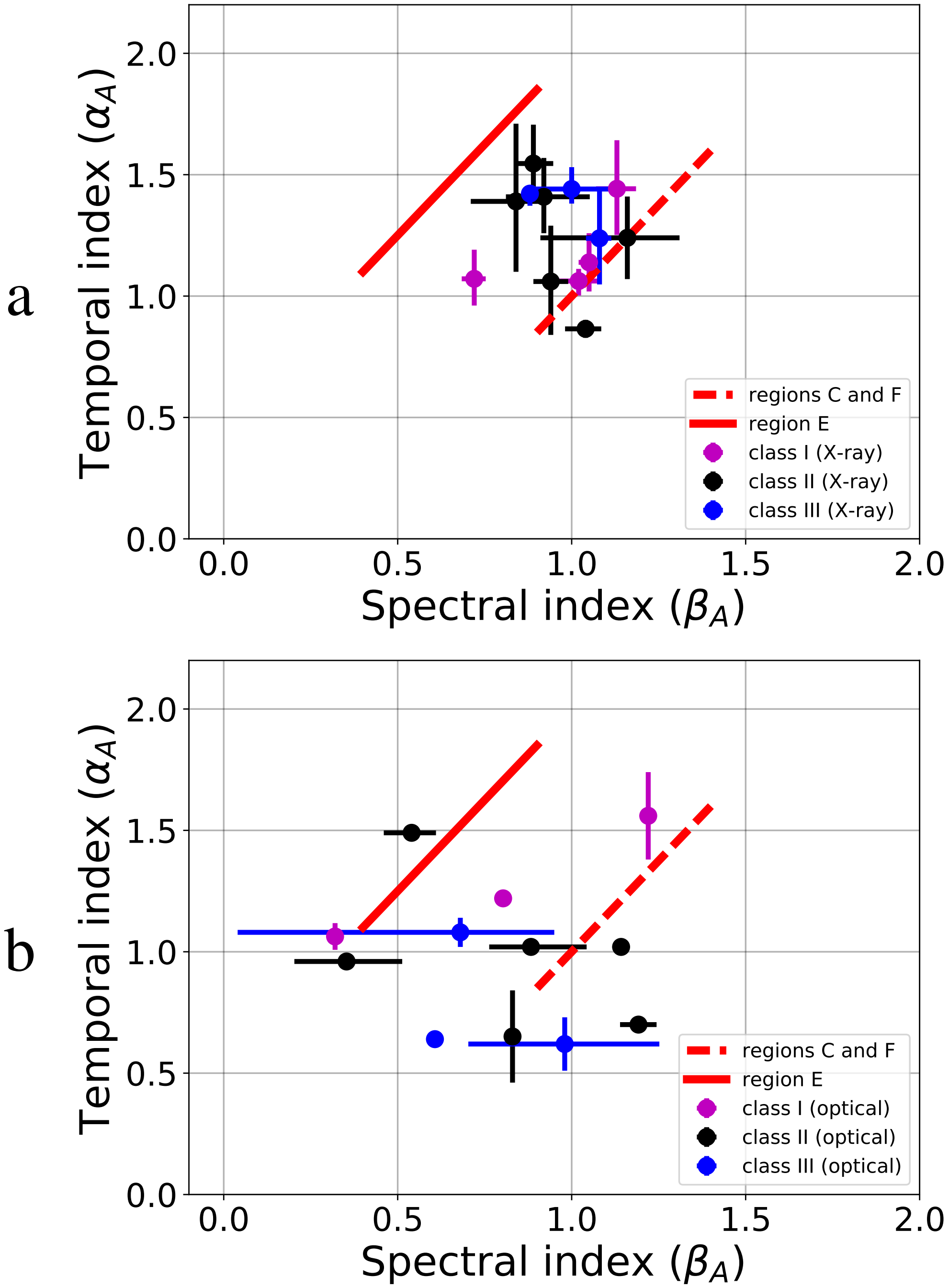


**Supplementary Figure 16. Temporal and spectral indices of the 13 GRBs during the self-similar phase (a) in the X-ray band and (b) in the optical band.** The spectral index in the optical band of 130831A (in class I) during the self-similar phase could not be retrieved from the literature, and therefore this GRB is excluded from panel **(b)**. The color coding is as in Supplementary Fig. 15 for the data points and regions. In the X-ray band, all the data points are within the expected theoretical limits of the closure relations of the model during this phase as well, while in the optical band, where the spectral indices are taken from the literature, the scatter is larger. The source data necessary to reproduce this Figure are provided as a Source Data file.

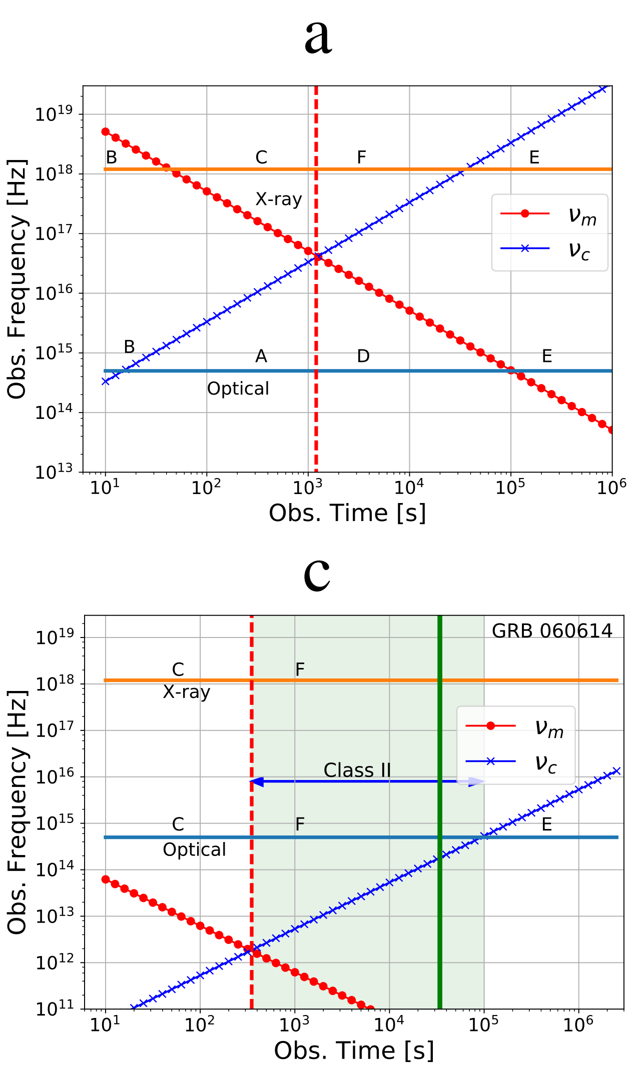


**Supplementary Figure 17. Temporal evolution of the injection frequency, $\nu_m$ (red points) and of the cooling frequency $\nu_c$ (blue crosses) in the coasting phase into a wind medium.** The letters (A, B, C, D, E, F) represent the six possible spectral and temporal regions given in Supplementary Table 7 and by Supplementary Equations (4) and (5). The orange and blue horizontal lines represent the X-ray and optical frequencies respectively. The red dashed vertical line indicates the crossing of the injection and of the cooling frequencies. It is clear that a given observed frequency can shift from one region to another only along one of the following paths: i) B-C-F-E or ii) B-A-D-E. Panel **(a)** is an illustration while panels **(b)**, **(c)** and **(d)** are three examples showing the occupied region (green vertical span) of the light curves of GRBs 080607, 060614, 050319 obtained from X-ray (in region F, F, E respectively) and optical data (in region E, F, E). The color coding is as in panel **(a)**, except that the green vertical line shows $T_{\mathrm{a,X}}$, the time marking the end of the plateau phase. The characteristic frequencies $\nu_m$ and $\nu_c$ are computed using the outflow parameters of GRBs 080607, 060614, 050319 presented in Table 3 (in the main manuscript). Clearly, during most of the plateau as well as the early afterglow phases, GRBs 080607, 060614, 050319 are classified as being in class I, II, III respectively.
